# C-Reactive Protein Enhances IgG-Mediated Cellular Destruction Through IgG-Fc Receptors *in vitro*

**DOI:** 10.3389/fimmu.2021.594773

**Published:** 2021-03-15

**Authors:** A. Robin Temming, Matthias Tammes Buirs, Arthur E. H. Bentlage, Louise W. Treffers, Hannah Feringa, Steven W. de Taeye, Taco W. Kuijpers, Sietse Q. Nagelkerke, Giso Brasser, Juk Yee Mok, Wim J. E. van Esch, Timo K. van den Berg, Theo Rispens, C. Ellen van der Schoot, Gestur Vidarsson

**Affiliations:** ^1^Sanquin Research and Landsteiner Laboratory, Department of Experimental Immunohematology, Amsterdam University Medical Center, University of Amsterdam, Amsterdam, Netherlands; ^2^Sanquin Research and Landsteiner Laboratory, Department of Blood Cell Research, Amsterdam University Medical Center, University of Amsterdam, Amsterdam, Netherlands; ^3^Sanquin Research and Landsteiner Laboratory, Department of Immunopathology, Amsterdam University Medical Center, University of Amsterdam, Amsterdam, Netherlands; ^4^Department of Pediatric Immunology, Rheumatology and Infectious Diseases, Amsterdam University Medical Center, Emma Children's Hospital, University of Amsterdam, Amsterdam, Netherlands; ^5^Sanquin Reagents, Sanquin, Amsterdam, Netherlands

**Keywords:** C-reactive protein, IgG, Fc receptor, FcγR, phagocytosis, cellular cytotoxicity, erythrocytes, tumor cells

## Abstract

Antibody-mediated blood disorders ensue after auto- or alloimmunization against blood cell antigens, resulting in cytopenia. Although the mechanisms of cell destruction are the same as in immunotherapies targeting tumor cells, many factors are still unknown. Antibody titers, for example, often do not strictly correlate with clinical outcome. Previously, we found C-reactive protein (CRP) levels to be elevated in thrombocytopenic patients, correlating with thrombocyte counts, and bleeding severity. Functionally, CRP amplified antibody-mediated phagocytosis of thrombocytes by phagocytes. To investigate whether CRP is a general enhancer of IgG-mediated target cell destruction, we extensively studied the effect of CRP on *in vitro* IgG-Fc receptor (FcγR)-mediated cell destruction: through respiratory burst, phagocytosis, and cellular cytotoxicity by a variety of effector cells. We now demonstrate that CRP also enhances IgG-mediated effector functions toward opsonized erythrocytes, in particular by activated neutrophils. We performed a first-of-a-kind profiling of CRP binding to all human FcγRs and IgA-Fc receptor I (FcαRI) using a surface plasmon resonance array. CRP bound these receptors with relative affinities of FcγRIa = FcγRIIa/b = FcγRIIIa > FcγRIIIb = FcαRI. Furthermore, FcγR blocking (in particular FcγRIa) abrogated CRP's ability to amplify IgG-mediated neutrophil effector functions toward opsonized erythrocytes. Finally, we observed that CRP also amplified killing of breast-cancer tumor cell line SKBR3 by neutrophils through anti-Her2 (trastuzumab). Altogether, we provide for the first time evidence for the involvement of specific CRP-FcγR interactions in the exacerbation of *in vitro* IgG-mediated cellular destruction; a trait that should be further evaluated as potential therapeutic target e.g., for tumor eradication.

## Introduction

Individuals with immune cytopenias have deficiencies in one or more blood cell types due to destruction by T-cells and/or myeloid and natural killer (NK) cells through opsonization by antibodies that target blood cell antigens. In immune-thrombocytopenic or -hemolytic patients, respectively, thrombocytes or erythrocytes are destroyed through autoantibodies (immune thrombocytopenia [ITP] and autoimmune hemolytic anemia [AIHA]) or alloantibodies (fetal or neonatal alloimmune thrombocytopenia [FNAIT], or hemolytic disease of the fetus or newborn [HDFN] and adverse transfusion reactions). These pathological antibodies comprise mainly immunoglobulin M (IgM) and IgG. Unfortunately, antibody levels often do not strictly correlate with either disease severity or clinical outcome (1–3) hinting at the involvement of additional factors.

Recently, we discovered that elevation of the acute-phase reactant C-reactive protein (CRP) in thrombocytopenic patients holds a prognostic value for ITP (4). The initial IgG-mediated myeloid activity initiated respiratory burst activity, which was a prerequisite for CRP opsonization. Functionally, CRP enhanced antibody-dependent cellular phagocytosis (ADCP) and release of reactive oxygen species (ROS) through respiratory burst responses toward IgG1-opsonized thrombocytes by phagocytes *in vitro*. In addition, administration of CRP, in combination with anti-thrombocyte IgG, decreased thrombocyte counts in mice. Later, these findings have been confirmed by others (5, 6). In humans, intravenous Ig (IVIg) treatment led to reduction in CRP levels that correlated with increased thrombocyte counts and decreased bleeding (4), and has also been shown to reduce leukocyte FcγR expression levels (7). Also in patients suffering from transfusion-related acute lung injuries (TRALI), CRP was found to be increased and to aggravate TRALI *in vivo* in mice (8, 9). Missing from these studies, however, was a functional link explaining how CRP mediates these enhanced effector functions and through which receptors.

CRP, a classical acute-phase protein, is mainly expressed by hepatocytes upon stimulation by pro-inflammatory cytokines, including interleukin (IL)-1, IL-6 and tumor necrosis factor α. During infections (including COVID-19) (10–13) and inflammatory conditions basal serum levels (<1 μg/ml) can be elevated up to 1,000-fold in a short time frame (14), making it a useful biomarker and in some cases even a predictor for disease progression. CRP belongs to the pentraxin family and has an annular homopentameric structure consisting of non-covalently linked protomers (15). Via the ligand-binding side, the B-face, CRP functions as an innate pattern recognition opsonin of microbial pathogens (16), oxidized low density lipoprotein (17), oxidized thrombocytes (8), and apoptotic cells (17, 18) through calcium-dependent binding to exposed phosphorylcholine (PC) residues.

The other side, the A-face, binds the C1q globular head domain (19–21) and is thereby able to trigger destruction via the classical complement pathway. In addition, several groups reported another destruction mechanism through receptors for the IgG-Fc (FcγRs) which have been proposed as binders of the CRP A-face with a 1:1 stoichiometry based on the co-crystal structure of homologous pentraxin serum amyloid component P with FcγRIIa (22). However, the answer to the question if FcγRs, and then which, bind CRP has remained elusive until recently. Some groups demonstrated binding of all classes (14, 22), but others designated the high-affinity IgG-Fc receptor FcγRIa (23) and/or low-affinity FcγRIIa (24) (with high affinity binding of the R131 [or p.166Arg] variant) as the main CRP receptors (25–27). Yet, others suggested that CRP is not a ligand for FcγRs and therefore other receptors must be responsible for its biological effect (28–31). Part of this discrepancy has been shown to be attributable to the cross-reactivity of mouse IgG(1) anti-CRP to human FcγRs on myeloid cells resulting in possible misinterpretation such as preferential binding of CRP to the R131 polymorphic variant of FcγRIIa (27, 30, 32). This emphasizes the importance of using antibody-free (e.g., Fab-based), and preferentially label-free, approaches to study CRP-FcγR interactions. Eventually, studies using such antibody-free platforms confirmed CRP binding to FcγRIa (23, 26, 33, 34), FcγRIIa (22, 23, 26, 34, 35), FcγRIIb (22), and FcγRIIIa (22), providing undisputable evidence for these receptors being CRP binders. Next to FcγRs, Lu et al. (36, 37) also identified the receptor for IgA-Fc (FcαRI) as another CRP receptor using an antibody-free surface plasmon resonance (SPR) platform. Importantly, a comprehensive study on the biochemical and functional properties of CRP-FcγR and FcαRI interactions, including allelic polymorphic variants of FcγR, has never been fully performed. Another gap in our current knowledge are the exact molecular bases of CRP interactions with all different FcγRs. However, the literature strongly suggests FcγR's first and second extracellular domain (D1 and D2, respectively) to be involved in CRP binding where the FcγR is proposed to dock into CRP's central pore interacting with two opposite protomers (14, 22, 23). IgG is also known to bind FcγR D2 (38), which likely forms the basis for the reported competition between CRP and IgG for FcγR binding (22, 23). In addition, the motifs on CRP involved in binding to FcγRIa, FcγRIIa and C1q show substantial homology to those found on IgG and mutagenesis screening demonstrated that binding sites on CRP for these FcγRs and C1q overlap (39). Taken together, current knowledge suggests that CRP binds to different types of FcγRs in a similar fashion and also overlapping with IgG binding sites. Nevertheless, detailed structural information is still lacking.

CRP's potential role in inflammation, atherosclerosis and heart disease is well-known, but detailed molecular information on the working mechanism is often lacking. This is certainly true in the context of antibody-mediated blood cell destruction. In patients with AIHA, antibody-opsonized erythrocytes are destroyed either directly through complement and/or Fc- and complement receptors on myeloid cells. Recent work of Meinderts et al. (40) suggests that basically all types of splenic myeloid cells (monocytes, macrophages, and polymorphonuclear leukocytes (PMNs; consisting mostly of neutrophils but also eosinophils and basophils)] play a role in the phagocytosis of IgG-opsonized erythrocytes. In AIHA, this tends to occur under inflammatory conditions (e.g., elevated IL-33, a positive regulator of IL-6 expression) (41, 42) which often result in episodes of increased CRP levels (43–47), although the causal link has not been established. Nevertheless, a recent case study of AIHA suggested CRP elevation to aggravate the anemia (48).

To dissect its working mechanism and to investigate whether CRP is a general enhancer of cellular destruction, we extensively investigated the influence of CRP on *in vitro* IgG-mediated erythrocyte destruction focusing on FcγR-mediated effector functions performed by different effector cell types. In addition, CRP-FcR interactions and their involvement in erythrocyte destruction were investigated thoroughly. Furthermore, we also studied whether CRP can be used to amplify tumor cell destruction by monoclonal antibodies (mAbs). Altogether, current study provides evidence for CRP as an enhancer of IgG-mediated phagocytosis and killing of erythrocytes, with a crucial role for CRP-FcγR interactions.

## Materials and Methods

### Antibodies and Human Fc Receptors

Monoclonal human anti-Rhesus D (RhD) IgG1, anti-RhD IgG2, and afucosylated anti-RhD IgG1 were produced as previously described by Temming et al. (49). Mouse IgG1 anti-CD64, anti-CD89, and isotype antibodies were deglycosylated through enzymatic digestion by incubating formulations in phosphate-buffered saline (PBS) for 2 h at 37°C with recombinant EndoS in an enzyme:substrate ratio of 1:20.

C-terminally biotinylated human FcγRIIIb-NA1 FcγRIIIb-NA2 and FcαRI were produced as follows: DNA sequences encoding the extracellular domain with an additional C-terminal tail containing, respectively, a linker, polyhistidine (10xHis)-tag and AVI-tag (GLNDIFEAQKIEWHE) were codon-optimized using GeneArt Tools (Invitrogen), ordered at Integrated DNA Technologies and cloned into the pcDNA3.1 mammalian expression vector. The receptors were produced in human embryonic kidney (HEK293) Freestyle cells as described previously (50). Five days after transfection, cell supernatants were harvested, filtered through a 0.2 mm filter and isolated through affinity chromatography on an ÄKTAprime plus system (GE Life Sciences) using a His-trap column (GE Life Sciences) according to manufacturer's protocol. Site-specific C-terminal BirA-mediated biotinylation was performed as described previously (51) with some adaptations: for biotinylation of 1 μM FcR, 3.3 nM BirA ligase was added and Amicon Ultra centrifugal filter units (MWCO 10 kDa) (Merck, Millipore) were used to concentrate the sample and to remove unbound biotin.

All purchased antibodies, Fab/F(ab')_2_ fragments and receptors are depicted in Table 1.

**Table 1:** Overview of the used commercial antibodies and other reagents.

**Name**	**Conjugate**	**Clone**	**Product**	**Firm**	**Application**
Mouse anti-CD64 IgG1	Biotin	10.1	555526	BD Pharmigen	F
Streptavidin	APC	N.A.	405207	BioLegend	F
Mouse anti-CD89 IgG1	N.A.	MIP7C	MA1-72500	Invitrogen	R,P,C
Mouse IgG1 isotype	N.A.	203	M1451	Pelicluster	R,P,C
anti-CD64 F(ab′)_2_	N.A.	10.1	216–520	Ancell	R,P,C
anti-CD32 F(ab′)_2_	N.A.	7.3	181–520	Ancell	R,P,C
anti-CD16 Fab	N.A.	3G8	165–580	Ancell	R,P,C
Mouse IgG1 isotype F(ab′)_2_	N.A.	MOPC31C	278–520	Ancell	R,P,C
FcγRIa	Biotin	N.A.	10256-H27H-B	Sino Biological	S
FcγRIIa-H131	Biotin	N.A.	10374-H27H1-B	Sino Biological	S
FcγRIIa-R131	Biotin	N.A.	10374-H27H-B	Sino Biological	S
FcγRIIb	Biotin	N.A.	10259-H27H-B	Sino Biological	S
FcγRIIIa-V158	Biotin	N.A.	10389-H27H1-B	Sino Biological	S
FcγRIIIa-F158	Biotin	N.A.	10389-H27H-B	Sino Biological	S

*F, flow cytometry; R, respiratory burst assay; P, phagocytosis assay; C, cellular cytotoxicity assay; S, SPR; N.A., not applicable*.

### Cells

All types of effector cells used in this study were derived from peripheral blood voluntary donated by anonymized healthy individuals (57% female, ages ranging from 24 to 63 with a median of 53 [first and third quartiles were 41 and 58, respectively]). Monocytes and NK cells were isolated from heparinized whole blood through Ficoll Hipaque gradient centrifugation. The peripheral blood mononuclear cell ring fractions were collected washed twice in PBS supplemented with 10% TNC and resuspended in magnetic-activated cell sorting (MACS) buffer (PBS supplemented with 10% TNC and 0.5% human serum albumin). After centrifugation, cells were resuspended in MACS buffer (85 μl per 10 × 10^6^ cells). For NK cell isolation, 15 μl CD56 MicroBeads (Miltenyi Biotec) were added per 10 × 10^6^ cells and for monocytes CD14 MicroBeads (Miltenyi Biotec). Cells were allowed to bind to the beads for 20 min on ice. After two cold washing steps in MACS buffer, cell pellet was resuspended in 5 ml MACS buffer and added to an LS column which was then washed three times with 3 ml MACS buffer. Five milliliters MACS buffer was added and purged into a 14 ml tube.

PMN fractions were isolated from the Ficoll Hipaque pellet through two rounds of erythrocyte lysis at 4°C with hypotonic lysis buffer (150 mM NH_4_Cl, 10 mM KHCO_3_, 0.1 mM ethylenediaminetetraacetic acid [EDTA], pH 7.2–7.4). After centrifugation, PMNs were resuspended in Iscove's Modified Dulbecco's Medium (IMDM; Gibco) supplemented with 10% heat-inactivated fetal calf serum (FCS) at 5×10^6^ cells per ml and were used directly in functional assays (resting/unstimulated/unprimed PMNs, referred to as naïve PMNs hereafter) or first stimulated overnight (activated PMNs). For this stimulation, 50 ng/ml recombinant interferon gamma (IFN-γ; PeproTech Inc) and 10 ng/ml clinical grade granulocyte colony-stimulating factor (G-CSF; Neupogen; Amgen) were added to the cells and incubated in an incubator overnight at 37°C and 5% CO_2_, as described previously (52, 53). After overnight incubation, cells were washed and resuspended in fresh IMDM without IFN-γ and G-CSF. Donor genotyping for *FCGR2A-*H/R131 polymorphism (rs1801274) was performed by multiplex ligation-dependent probe amplification as described previously (54).

RhD-positive erythrocyte target cells (donor 18-1000 with R2R2 [*DcE/DcE*] genotype) were provided by the department of erythrocyte serology. Her2-positive human breast cancer cells from the SKBR3 cell line (ATCC) were cultured in IMDM supplemented with 20% FCS, Penicillin-Streptomycin and 2 mM L-glutamine at 37°C and 5% CO_2_.

Before application, cells were always counted in an automated CASY cell counter and analyzer (Roche) to determine cell numbers and viability (> 90%) to make sure accurate cell concentrations were used in the functional assays.

### Phagocytosis and Respiratory Burst Assay

Target cells were fluorescently labeled using the PKH26 Red Fluorescent Cell Linker Kit (PKH26GL; Sigma-Aldrich) according to manufacturer's protocol and incubated in the dark for 4 min at room temperature, followed by two washing steps in PBS. Subsequently, 5 × 10^5^ erythrocytes were pre-opsonized in a 96-wells V-bottom plate with IgG diluted in reaction buffer (HEPES buffer [20 mM HEPES, 132 mM NaCl, 6 mM KCl, 1 mM MgSO_4_, 1.2 mM H_2_PO_4_] supplemented with 3 mM CaCl_2_ and 0.5% bovine serum albumin [BSA]) in the dark for 30 min at room temperature on a shaker. After opsonization, plates were washed twice with reaction buffer by centrifugation (1,800 rpm for 2 min) to remove unbound IgG. Effector cells diluted in reaction buffer supplemented with or without native CRP (from human fluid; Sigma-Aldrich) were added to the pre-opsonized erythrocytes in an effector-to-target (E:T) ratio of 1:1 and incubated in the dark on a shaker (175 rpm) for 45 min at 37°C. The reaction was stopped by keeping the plates on ice and non-ingested erythrocytes were lysed by concomitant centrifugation (1,800 rpm for 2 min) and incubation in cold erythrocyte lysis buffer. After another lysis step, plates were centrifuged and samples were resuspended in PBS. Phagocytosis was measured through flow cytometry using an LSRII with a high-throughput sampler (BD Biosciences) and depicted as geometric mean fluorescence intensities (gMFI) or as an index of the number of erythrocytes per 100 effector cells, calculated with the following equation: phagocytic index = (gMFI_positive PMNs_ ÷ gMFI_erythrocytes_) × percentage_positive PMNs_. All conditions were measured at least in duplicate.

For respiratory burst assays the same setup was used where 10 μM dihydrorhodamine-1,2,3 (DHR-1,2,3; Thermo Fisher Scientific) was added to the effector cells just before the reaction in order to monitor effector cell nicotinamide adenine dinucleotide phosphate (NADPH) oxidase activity through the oxidation-induced conversion of DHR-1,2,3 to fluorescent Rhodamine-1,2,3. To study calcium dependency, 5 mM EDTA diluted in reaction buffer was added to the reaction. Similarly, 10 μM diphenylene iodonium (DPI [broad-spectrum inhibitor of flavoproteins, including NADPH oxidase machinery]; Sigma-Aldrich) was added to the reaction buffer to study oxidation dependency.

In FcR blocking experiments, effector cells (10 × 10^6^ per ml) were pre-incubated in reaction buffer containing respective blocking agents (each 10 μg/ml) for 45–50 min, at room temperature, prior to addition to the pre-opsonized target cells.

### Cytotoxicity Assay

Erythrocytes (100 × 10^6^) were labeled with 100 μCi Chromium-51 (Cr-51; Perkin-Elmer) for 1 h at 37°C as previously described by Temming et al. (49). Consecutively, effector cells (50 μl of 4 × 10^6^ per ml for monocytes and PMNs, 1 × 10^6^ per ml for NK cells) and radioactive erythrocytes (25 μl of 4 × 10^6^ per ml) were added to antibody/CRP mixtures (35 μl) to an end volume of 110 μl IMDM 10% FCS and an E:T ratio of 2:1 for monocytes and PMNs and 1:2 for NK cells. Subsequently, the 96-wells V-bottom plates were centrifuged for 1 min at 1,317 rpm to pellet the cells and incubated for 4 h at 37°C and 5% CO_2_. For FcR blocking experiments the same setup was used where effector cells were pre-incubated with blocking agents (each 10 μg/ml) in IMDM 10% FCS for 45–50 min at room temperature.

Cytotoxicity assays with the SKBR3 cells as target were performed as described previously by Treffers et al. (52). In brief, Cr-51-labeled SKBR3 cells (50 μl of 1 × 10^5^ per ml) were added to stimulated PMNs (50 μl of 5 × 10^6^ per ml) in an E:T ratio of 50:1 and end volume of 100 μl RPMI supplemented with 10% FCS, Penicillin-Streptomycin and 2 mM L-glutamine containing 10 μg/ml trastuzumab/Herceptin in the presence or absence of 10 μg/ml CRP. Subsequently, the 96-wells U-bottom plates were centrifuged for 2 min at 1,317 rpm to pellet the cells and incubated for 4 h at 37°C and 5% CO_2_.

After the reaction, plates were centrifuged for 5 min at 1,317 rpm, the supernatant was harvested and transferred into transfer tubes which were measured in a Gamma Counter machine and erythrocyte or SKBR3 lysis was calculated using the following formula: Cytotoxicity = ([counts_sample_–counts_background_] ÷ [counts_100%_–counts_background_]) × 100%. All conditions were measured in triplicate.

### Surface Plasmon Resonance Imaging

SPR measurements were performed on an IBIS MX96 (IBIS technologies) as described previously by Dekkers et al. (55). All C-terminally biotinylated FcRs were spotted using a Continuous Flow Microspotter (Wasatch Microfluidics) onto a single SensEye G-streptavidin sensor (1–08–04–008, Ssens) allowing for binding affinity measurements of CRP to all FcRs simultaneously on the IBIS MX96. The biotinylated FcRs were spotted in 3-fold dilutions, ranging from 30 nM to 1 nM for FcγRIIa-p.His166Arg (H/R131), FcγRIIb, FcγRIIIa-p.176Phe (F158), FcγRIIIb-NA1/NA2, and ranging from 100 nM to 3nM for FcγRIIIa-p.176Val (V158) and FcαRI. Biotinylated FcγRI was spotted using the following concentrations: 100, 30, 10, 6, 3 and 1 nM. All FcRs were spotted in PBS supplemented with 0.075% Tween-80 (M126–100 ml; VWR), pH 7.4. After spotting, the sensor was blocked with 200 nM biotinylated BSA (A8549; Sigma-Aldrich) for 7 min. Subsequently, human CRP was injected at 2-fold dilution series ranging from 0.98 nM to 2,000 nM in 20 mM Tris buffer supplemented with 280 mM NaCl and 5 mM CaCl_2_, pH8.0. To confirm receptor functionality, 1,000 nM of monoclonal human IgG1 and IgA1 were flowed over the sensor. Regeneration after each sample was carried out with 10 mM Gly-HCl, pH 2.4. Calculation of the dissociation constant (K_D_) was performed by equilibrium fitting to R_max_ = 500.

### FcγRIa Expression

Effector cell FcγRIa expression levels were determined by incubating cells with 1:25-diluted biotin-conjugated anti-CD64 in 50 μl PBS for 1 h at 4°C. After two washing steps in PBS by centrifugation (1,800 rpm for 2 min), cells were incubated with 1:200-diluted allophycocyanin (APC)-conjugated streptavidin in 50 μl PBS for 20 min at 4°C. Subsequently, cells were washed twice in PBS by centrifugation, resuspended in 200 μl PBS and fluorescence was measured using an LSRII with a high-throughput sampler. Gating strategy is depicted in Supplementary Figure 1.

### Data Processing and Analysis

Flow cytometry data was processed using FlowJo software (FlowJo LLC) and Excel. SPR data was processed using SprintX software (IBIS Technologies) and analysis and calculation of binding data was carried out with Scrubber software version 2 (Biologic Software) and Excel. Statistical analyses were performed and all graphs were generated using GraphPad Prism 8.0.1 (GraphPad Software Inc.).

## Results

### CRP Potentiates IgG1-Mediated Respiratory Burst Activity Toward Opsonized Erythrocytes

**Figure 1:**
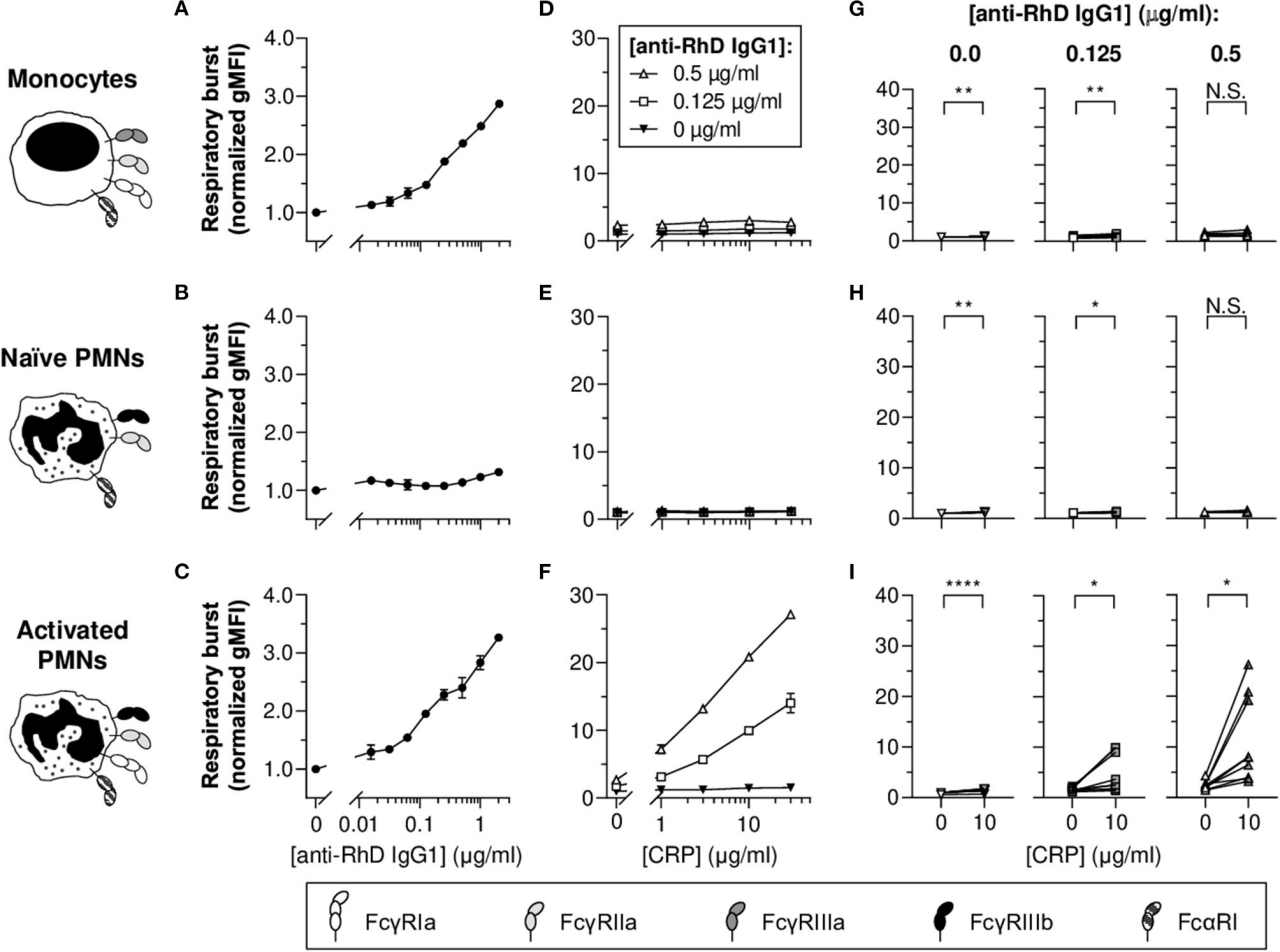
CRP potentiates IgG1-mediated respiratory burst activity of activated PMNs toward opsonized erythrocytes. **(A–C)** IgG1 concentration-dependent respiratory burst activity (normalized geometric mean fluorescence intensities [gMFI] ± standard error of the mean [S.E.M.]; signals of the 0 μg/ml IgG1 condition were set to 1.0) of monocytes **(A)**, naïve PMNs **(B)**, and G-CSF/IFN-γ-stimulated PMNs **(C)** toward pre-opsonized target erythrocytes. **(D–F)** Titration curves of CRP dilutions (30, 10, 3, 1, 0 μg/ml) added to the reaction with different anti-RhD IgG1 backgrounds (0.5 μg/ml, white triangles; 0.125 μg/ml, white squares; 0 μg/ml, black triangles) using effector cells from the same donor as in **(A–C)**. **(A,D; B,E; C,F)** Depict data from one effector cell donor representative for 5–9 donors tested in 3–5 independent experiments. **(G–I)** The impact of CRP (10 μg/ml) on respiratory burst activity triggered by 0, 0.125, and 0.5 μg/ml anti-RhD IgG1 using effector cells from different donors. Schematic representations of the effector cells indicate the types of FcγRs and FcαRI expressed by these cells (52, 56–58) but do not reflect relative expression levels. Two-tailed paired *t-*test was used to determine significant differences. *p ≤ 0.05; **p < 0.01; ****p < 0.0001; N.S. not significant.

First, we investigated the potency of monocytes and PMNs to exert respiratory burst activity toward opsonized erythrocytes (Figure 1). For this purpose, monoclonal human anti-RhD IgG1 (exhibiting 94% core fucosylation) was produced and effector cells were labeled with DHR-1,2,3 to measure NADPH oxidase activity using flow cytometry. Results show IgG1 concentration-dependent respiratory burst activity toward opsonized erythrocytes by monocytes, but not naïve PMNs (Figures 1A,B, respectively). PMNs only showed respiratory burst activity toward opsonized erythrocytes after stimulation with IFN-γ and G-CSF mimicking inflammatory conditions (Figure 1C), causing PMN activation and upregulation of FcγRIa (Supplementary Figure 1). In addition, this treatment slightly decreased FcγRIIa levels on PMN surfaces, as well as FcγRIIIb levels (prone to shedding), which is in full agreement with previous studies (52, 56) (Supplementary Figure 1), whilst expression of FcαRI is known to be unaffected (57).

To determine whether CRP affects respiratory burst responses of PMNs and monocytes toward erythrocytes, physiologically relevant concentrations (30–1 nM) of native human CRP were titrated into the reaction buffer in the presence or absence of anti-RhD IgG1 (Figures 1D–I and Supplementary Figure 2). Varying CRP was added at indicated concentration of IgG1 within the titratable range (Figures 1A,C). In general, for monocytes and naïve PMNs, CRP-mediated enhancement was negligible or absent. For activated PMNs, CRP significantly enhanced IgG1-mediated respiratory burst activity in a concentration-dependent manner (Figures 1F,I and Supplementary Figure 2), similar to what we found previously for thrombocytes (4). In the absence of IgG, CRP also significantly enhanced respiratory burst activity by activated PMNs, but the magnitude of this effect was minimal (from 1.0 ± 0.03 to 1.5 ± 0.07 normalized gMFI; *p* < 0.0001), whilst this was substantial in the presence of 0.125 (from 1.6 ± 0.1 to 4.7 ± 0.9 normalized gMFI; *p* = 0.0290) and 0.5 μg/ml IgG (from 2.4 ± 0.2 to 11.1 ± 2.0 normalized gMFI; *p* = 0.0114) (Figure 1I [see Supplementary Figure 2 for other CRP concentrations]).

### CRP Enhances IgG1-Mediated Erythrophagocytosis by Activated PMNs

**Figure 2:**
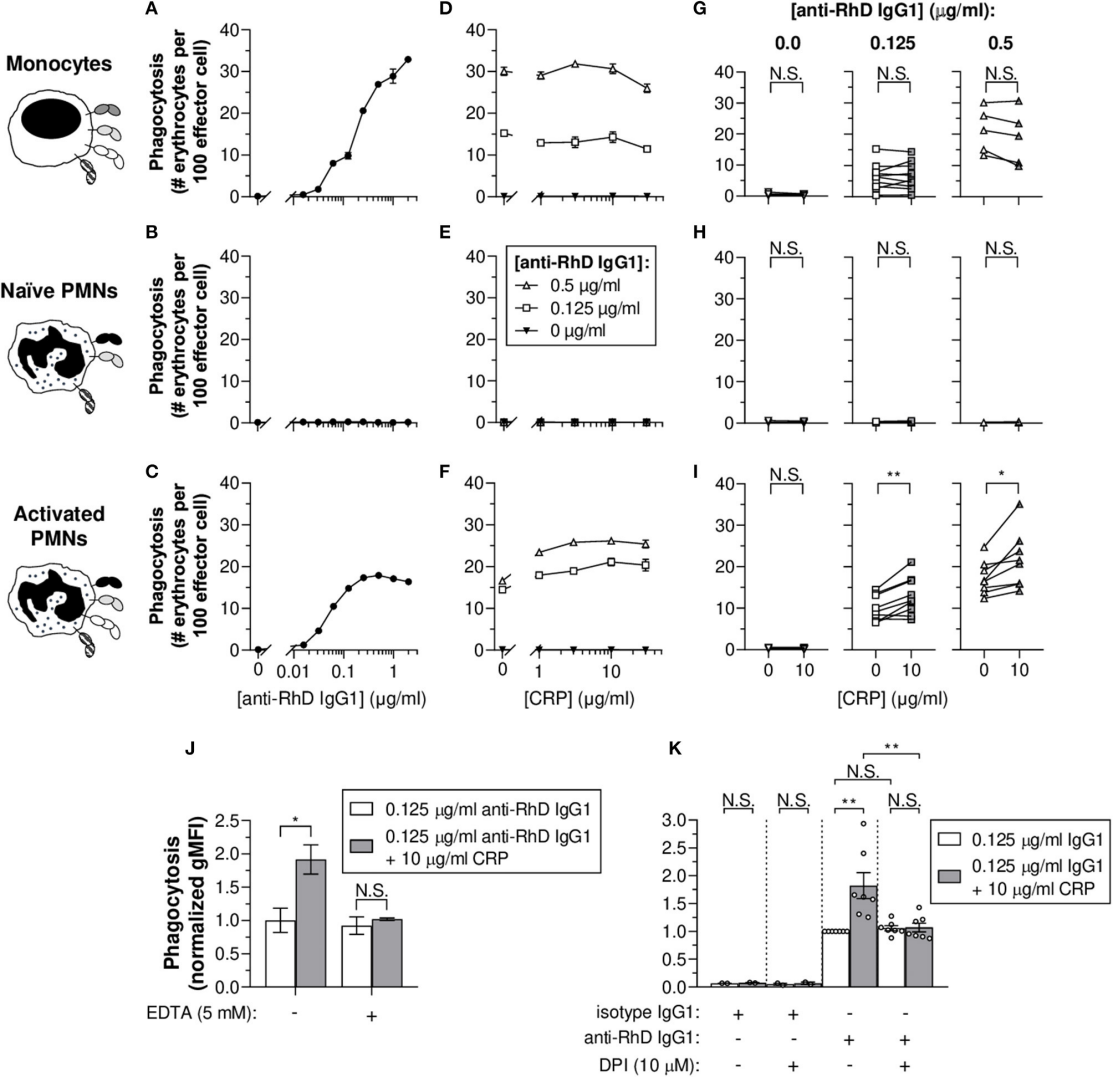
CRP boosts IgG1-mediated erythrophagocytosis by activated PMNs and is calcium- and respiratory burst-dependent. **(A–C)** IgG1 concentration-dependent phagocytosis (phagocytic index as number [#] of erythrocytes per 100 effector cells ± S.E.M.) of monocytes **(A)**, naïve PMNs **(B)**, and G-CSF/IFN-γ-stimulated PMNs **(C)** toward pre-opsonized target erythrocytes. **(D–F)** Titration curves of CRP dilutions (30, 10, 3, 1, 0 μg/ml) added to the phagocytosis reaction with different anti-RhD IgG1 backgrounds (0.5 μg/ml, white triangles; 0.125 μg/ml, white squares; 0 μg/ml, black triangles) using effector cells from the same donor as in **(A–C)**. **(A,D; B,E; C,F)** Depict data from one effector cell donor representative for 5–9 donors tested in 3–5 independent experiments. **(G–I)** The impact of CRP (10 μg/ml) on phagocytosis triggered by 0, 0.125, and 0.5 μg/ml anti-RhD IgG1 using effector cells from different donors. Schematic representations of the effector cells indicate the types of FcγRs and FcαRI expressed by these cells (52, 56–58) but do not reflect relative expression levels. **(J)** Calcium dependency of the enhancing CRP effect on phagocytosis (normalized gMFI ± S.E.M.; signals of the 0.125 μg/ml anti-RhD IgG1 condition without CRP were set to 1.0) by G-CSF/IFN-γ-stimulated PMNs was studied by adding chelator EDTA (5 mM) to the reaction (0.125 μg/ml anti-RhD or isotype IgG1) in the presence (gray bars) or absence (white bars) of 10 μg/ml CRP. **(K)** The dependency of the CRP effect on effector cell respiratory burst response was studied by adding oxidation inhibitor DPI (10 μM) to the phagocytosis reaction (normalized gMFI ± S.E.M.) using G-CSF/IFN-γ-stimulated PMNs from different donors (depicted as individual data points). Two-tailed paired *t-*test was used to determine significant differences in panels **(G–I)** and One-way ANOVA for **(J,K)**. *p ≤ 0.05; **p < 0.01; N.S. not significant.

To study the potency of monocytes and PMNs to exert phagocytosis of IgG-opsonized erythrocytes, target cells were fluorescently labeled. Flow cytometry data demonstrate that monocytes were able to perform IgG1 concentration-dependent erythrophagocytosis (Figure 2A). PMNs also phagocytosed erythrocytes in a concentration-dependent manner but only after stimulation with G-CSF and IFN-γ (Figures 2B,C). In accordance to the broad individual variation (Figure 1I and Supplementary Figure 2), the G-CSF and IFN-γ treatment of PMNs inflicted a broad range of FcγRIa expression levels which appeared to correlate to some extent with the magnitude of phagocytosis (R^2^ = 0.188 and *p* = 0.0065) (Supplementary Figure 3), underscoring the potential importance of FcγRIa in PMN erythrophagocytosis. In addition, blocking of FcγRIa and to a lesser extent FcγRIII decreased phagocytosis responses by monocytes (from 13.3 ± 2.0 to 0.5 ± 0.1 and 6.6 ± 2.2 # erythrocytes per 100 effector cells, respectively; *p* = 0.0101 and *p* = 0.1966) and activated PMNs (from 17.7 ± 3.7 to 0.5 ± 0.1 and 7.4 ± 1.4 # erythrocytes per 100 effector cells, respectively; *p* = 0.0089 and *p* = 0.1578) (Supplementary Figure 4).

To determine whether CRP affects IgG-mediated (baseline) erythrophagocytosis of PMNs and monocytes, CRP was titrated into the reaction in the presence or absence of anti-RhD IgG1 (Figures 2D–I and Supplementary Figure 5). For activated PMNs (Figures 2F,I and Supplementary Figure 5), but not monocytes (Figures 2D,G and Supplementary Figure 5) nor naïve PMNs (Figures 2E,H), CRP addition resulted in significant enhancement of baseline phagocytic index in the presence of 0.125 (e.g., for 10 μg/ml CRP: from 10.2 ± 0.7 to 12.9 ± 1.1 # erythrocytes per 100 effector cells; *p* = 0.0035) or 0.5 μg/ml IgG1 (from 17.3 ± 1.0 to 21.6 ± 1.7 # erythrocytes per 100 effector cells; *p* = 0.0133). Similar stimulation of monocytes with G-CSF and IFN-γ only marginally upregulated their FcγRIa expression (Supplementary Figure 6). This did not enhance CRP-mediated potentiation of erythrophagocytosis by monocytes (Supplementary Figure 6).

### CRP-Mediated Potentiation of PMN Erythrophagocytosis Requires Calcium and Depends on Effector Cell Respiratory Burst Activity

To confirm the calcium-dependency of CRP opsonization, chelator EDTA was added to the phagocytosis reaction. As expected, addition resulted in abolishment of the CRP-mediated enhancement of erythrophagocytosis (from +0.9 ± 0.2 to +0.1 ± 0.2 normalized gMFI; *p* = 0.0479 and *p* = 0.9686), indicating the observed CRP effects depend on calcium (Figure 2J). In addition, CRP enhancement of PMN antibody-dependent respiratory burst required intact NADPH oxidase, as burst activity was significantly lowered in the presence of its inhibitor; DPI (a broad-spectrum inhibitor of flavoproteins, including NADPH oxidase machinery) (Supplementary Figure 7). DPI also abolished CRP-enhancing effects of erythrophagocytosis (from +0.8 ± 0.2 to +0.01 ± 0.16 normalized gMFI; *p* = 0.0014 and *p* > 0.9999) without affecting baseline ADCP (*p* > 0.9999) (Figure 2K). This indicates that the CRP amplification, but not IgG1-mediated phagocytosis, depends on effector cell respiratory burst activity.

### CRP Enhances IgG1-Mediated Lysis of Opsonized Erythrocytes by Activated PMNs

**Figure 3:**
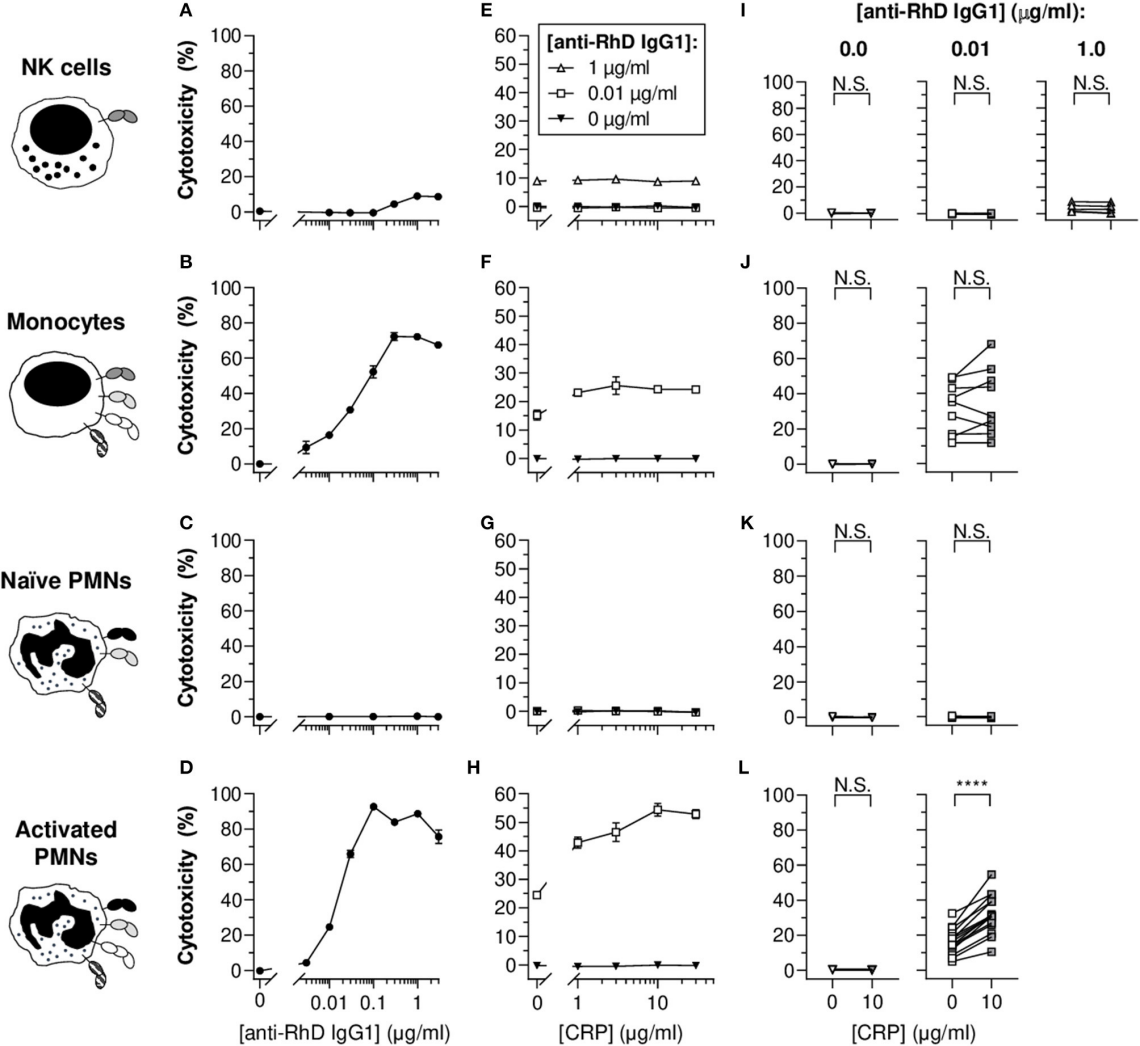
CRP enhances the IgG1-mediated cytotoxicity of opsonized erythrocytes by activated PMNs. **(A–D)** IgG1 concentration-dependent cytotoxic responses (% lysed target cells ± S.E.M.) of NK cells **(A)**, monocytes **(B)**, naïve PMNs **(C)**, and G-CSF/IFN-γ-stimulated PMNs **(D)** toward opsonized target erythrocytes. **(E–H)** Titration curves of CRP dilutions (30, 10, 3, 1, 0 μg/ml) added to the cytotoxicity reaction with different anti-RhD IgG1 backgrounds (1 μg/ml, white triangles; 0.01 μg/ml, white squares; 0 μg/ml, black triangles) using effector cells from the same donor as in **(A–D)**. **(A,E; B,F; C,G; D,H)** Depict data from one effector cell donor representative for 5–15 donors tested in 3–7 independent experiments. **(I–L)** The impact of CRP (10 μg/ml) on IgG1-mediated cytotoxicity using effector cells from different donors. The CRP effect was tested at 0 and 0.01 μg/ml anti-RhD IgG1 for all cells, but also at an additional higher dose at 1 μg/ml IgG1 for NK cells as indicated. Schematic representations of the effector cells indicate the types of FcγRs and FcαRI expressed by these cells (52, 56–58) but do not reflect relative expression levels. Two-tailed paired *t-*test was used to determine significant differences. ****p < 0.0001; N.S. not significant.

Since phagocytosis results in complete or partial target ingestion and respiratory burst activity does not necessarily reflect erythrocyte destruction, cellular cytotoxicity-mediated erythrocyte lysis was also investigated as a more direct measure of target cell fate. For this purpose, erythrocytes were radioactively labeled and three known antibody-dependent cellular cytotoxicity (ADCC)-performing cell types were included in the study: NK cells, monocytes and PMNs (Figures 3A–D). As expected, NK cells did not trigger potent ADCC responses toward the IgG1-opsonized target erythrocytes (Figure 3A), as full cytotoxic potential requires high-affinity interaction between IgG1 and FcγRIIIa acquired by afucosylation (18% core fucosylation) of the anti-RhD IgG1 (Supplementary Figure 8) (49, 59, 60). Subtle cytotoxicity (~5%) was detected at relatively high antibody concentrations (1–3 μg/ml). Similar to what we found for phagocytosis (Figures 2A–C), monocytes and activated PMNs triggered IgG1 concentration-dependent cytotoxicity, but naïve PMNs did not (Figures 3B–D). Addition of DPI dramatically confined IgG1-mediated cytotoxicity by activated PMNs (Supplementary Figure 9), which suggests that erythrocyte lysis at least partly depends on effector cell respiratory burst activity. The broad range of FcγRIa expression on activated PMNs induced by G-CSF/IFN-γ stimulation appeared to correlate with cytotoxic capacity (R^2^ = 0.7323 and *p* < 0.0001), suggesting a pivotal role for this receptor in IgG1-mediated PMN ADCC (Supplementary Figure 10) and also providing an explanation for the complete lack of cytotoxicity response for naïve PMNs which express negligible levels of FcγRIa (Supplementary Figure 1). Moreover, blocking of FcγRIa, and also FcγRII, resulted in decreased cytotoxic responses by monocytes (from 9.0 ± 0.7 to 1.1 ± 0.3 and 2.2 ± 0.5%, respectively; *p* < 0.0001 and *p* < 0.0001) and PMNs (from 40.7 ± 3.7 to 0.5 ± 0.4 and 11.7 ± 1.2%, respectively; *p* < 0.0001 and *p* < 0.0001) (Supplementary Figure 11).

CRP exclusively led to significant and concentration-dependent enhancement of cytotoxicity for monocytes and activated PMNs (Figures 3E–L and Supplementary Figure 12). The CRP-mediated enhancement of baseline cytotoxicity for monocytes was more subtle compared to PMNs, enhancing cytotoxicity for 5 out of 9 donor monocytes (Figure 3J, not significant; *p* = 0.2821), whilst for PMNs cytotoxicity was amplified in all tested donors with a relative mean increase of 104.2 ± 10.4% (*p* < 0.0001) in erythrocyte lysis (Figure 3L). In line with limited effect of G-CSF/IFN-γ treatment of monocytes on FcγRIa expression, CRP did not trigger enhanced potentiation of cytotoxicity compared to unstimulated equivalents (Supplementary Figure 6). Since CRP did not potentiate NK cell cytotoxic responses (Figure 3E and Supplementary Figure 12), even not in the presence of afucosylated IgG1 (Supplementary Figure 8), we hypothesized that if CRP functions through FcγRs, this would most likely not include FcγRIIIa (the only FcγR expressed by this cell type).

### CRP Binds Immobilized Human FcγRs and FcαRI

**Figure 4:**
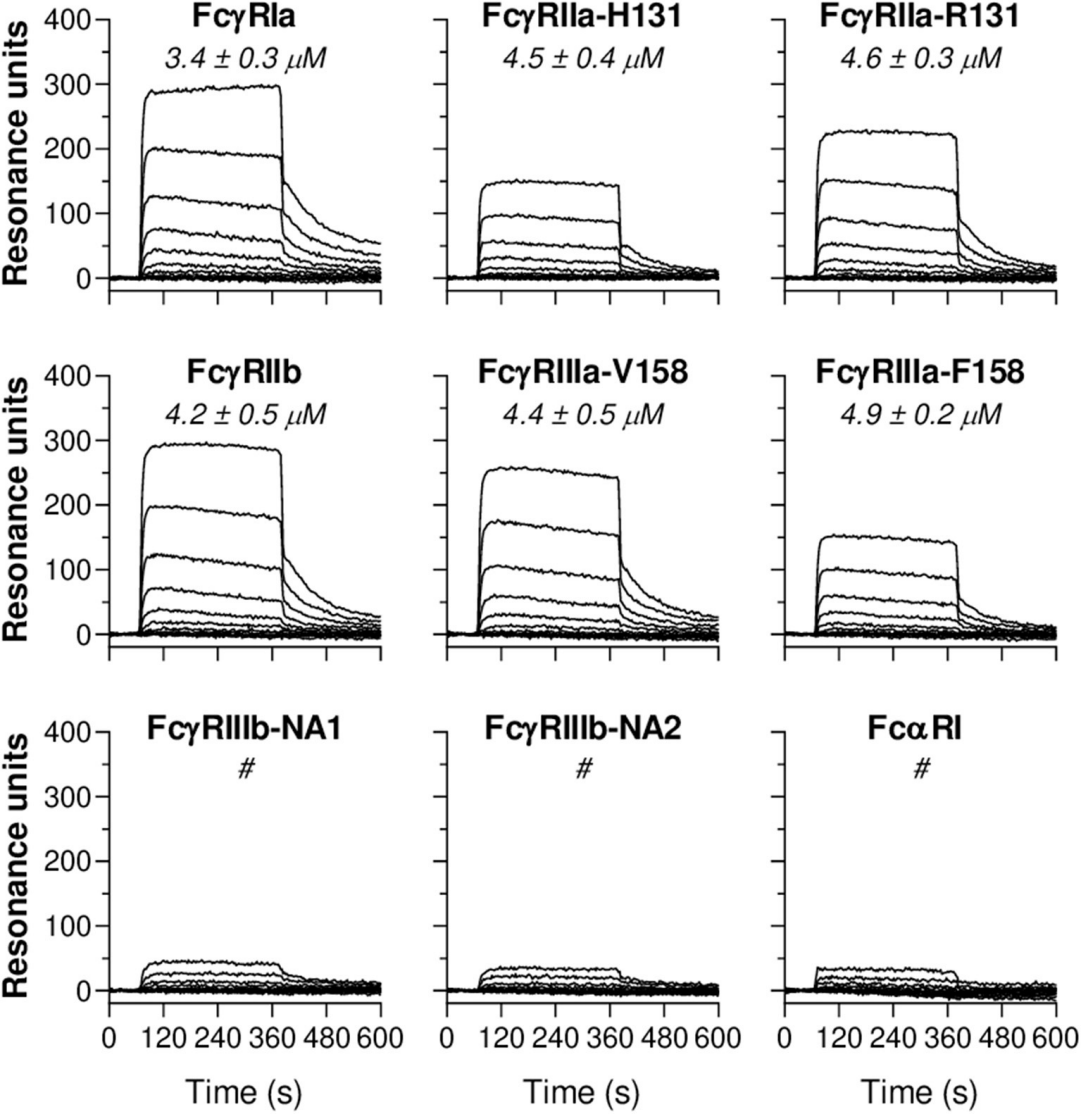
CRP binds immobilized human Fc receptors. Representative sensorgrams showing binding kinetics of human CRP to C-terminally immobilized human FcγR classes (FcγRIa, FcγRIIa-H/R131, FcγRIIb, FcγRIIIa-V/F158, FcγRIIIb-NA1/NA2) and FcαRI. Depicted data are representative for three independent experiments where CRP binding was imaged in real-time and simultaneously to FcRs spotted at different densities (100–1 nM). Each line represents the kinetics of a specific CRP dilution (1:1 serial dilutions ranging from 2,000 to 1 nM). For all receptors, in each sensorgram CRP's exact binding affinity is depicted by the mean K_D_ value (μM ± S.E.M., averaged from triplo data) at R_max_ of 500. ^#^ indicates binding of CRP to the receptor which was too low to calculate K_D_.

To study CRP-FcR interactions, C-terminally biotinylated FcγRs and FcαRI were immobilized onto a streptavidin-coated SPR array at different densities. Subsequently, serial dilutions of soluble CRP were flowed over this FcR array and binding kinetics were monitored in real-time. Resulting sensorgrams demonstrated that CRP binds immobilized FcγRIa, FcγRIIa/b, and FcγRIIIa (Figure 4). In addition, calculated K_D_ values show that these affinities fall within the same low affinity range (3.4–4.9 μM). Receptor density also seems to influence CRP binding strength since K_D_ values decreased (increasing affinity) with increasing R_max_ (Supplementary Figure 13). Particularly low binding was observed for FcγRIIIb (too weak to quantify), as well as for FcαRI (Figure 4), whereas binding patterns for IgA1 did show efficient binding (Supplementary Figure 14). Binding data also indicate that CRP does not bind differentially to polymorphic FcγR variants as affinities for FcγRIIa-H/R131 (or p.His166Arg) and FcγRIIIa-V/F158 (or p.Val176Phe) were very similar. Altogether, these findings demonstrate CRP's ability to bind FcγRs and FcαRI; a feature potentially involved in its enhancing effect on IgG-mediated effector functions we observed in Figures 1–3.

### FcR Blocking Affects IgG1-Mediated PMN Effector Functions and the Enhancing CRP Effect

**Figure 5:**
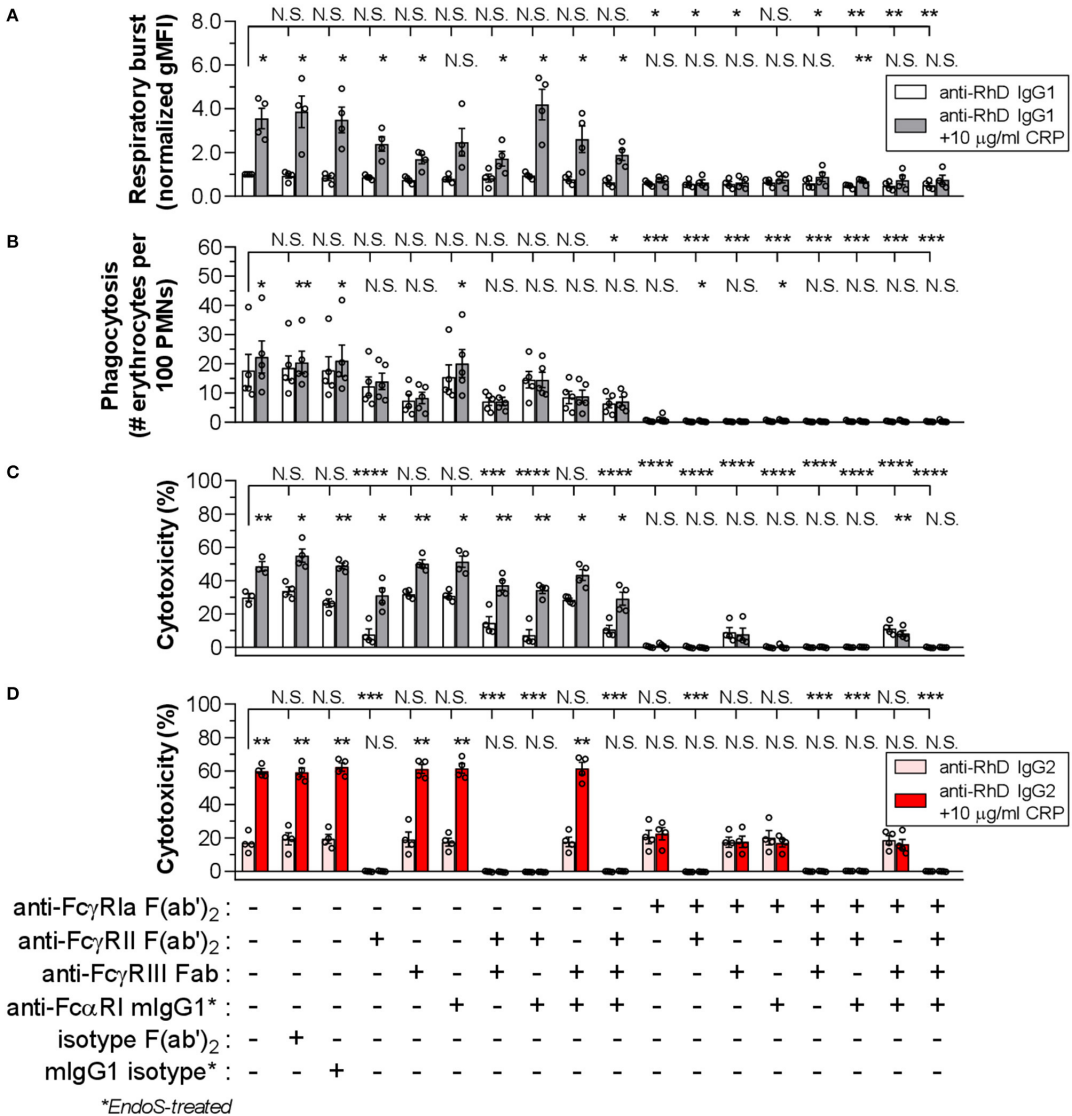
FcγRIa is mainly responsible for CRP-mediated enhancement of PMN effector functions. The role of FcRs in IgG1-mediated PMN respiratory burst activity **(A)**, phagocytosis **(B)**, and cytotoxicity **(C)** was determined using a panel of FcR blocking agents in the presence (gray bars) or absence (white bars) of CRP (10 μg/ml). For blocking, effector cells were pre-incubated with the respective blocking agent, or isotype equivalent for control purposes, at a concentration of 10 μg/ml. An IgG1 concentration of 0.125 μg/ml was used for respiratory burst and phagocytosis tests, and 0.01 μg/ml for cytotoxicity assay. For respiratory burst data, gMFI values from conditions without blocking and CRP were normalized to 1.0. Phagocytosis is depicted as phagocytic index (# erythrocytes per 100 neutrophils) and cytotoxicity as the percentage (%) of lysed target erythrocytes. Respiratory burst and cytotoxicity blocking experiments have been performed using G-CSF/IFN-γ-stimulated PMNs from 4 donors tested in three independent experiments and phagocytosis tests for 5 donors tested in four independent experiments. **(D)** Similarly, the role of FcRs in IgG2-mediated cytotoxicity was determined in the presence (red bars) or absence (pink bars) of CRP (10 μg/ml), using the same panel of FcR blocking agents and an IgG concentration of 0.5 μg/ml was used. IgG2-based cytotoxicity assays have been performed in three independent experiments using G-CSF/IFN-γ-stimulated PMNs from 4 FcγRIIa^H/H131^ donors. Bar graphs indicate the mean value ± S.E.M. of data points (circles) each representing the mean of duplo **(A,B)** or triplo **(C,D)** values. Significant differences between blocking conditions have been determined using one-way ANOVA and two-tailed paired *t-*test was used to determine significant differences between conditions with or without CRP. *p ≤ 0.05; **p < 0.01; ***p < 0.001; ****p < 0.0001; N.S. not significant.

To investigate whether CRP-FcR interactions are involved in the CRP enhancement of IgG-mediated effector functions, blocking experiments were performed using activated PMNs and a panel of FcR-blocking Fab/F(ab')_2_ fragments or deglycosylated mouse IgG1 (Figure 5). Looking first at IgG1-mediated respiratory burst and phagocytosis (Figures 5A,B, respectively), FcγRIa showed a clear dominance (*p* ≤ 0.0418 and *p* ≤ 0.0002, respectively), with a slight but not significant effect after blocking either FcγRII (*p* = 0.9760 and *p* = 0.7473), FcγRIII (*p* = 0.4331 and *p* = 0.0635), or in combination (*p* = 0.8718 and *p* = 0.0515). As expected, additional blocking of FcαRI had no significant effect (*p* ≥ 0.0899 and *p* ≥ 0.1314), except for phagocytosis in combination with FcγRII and FcγRIII blocking (*p* = 0.0328 [*p* = 0.0515 without FcαRI block]) (Figures 5A,B).

With the addition of CRP, a clear enhancing effect (3.6-fold [from 1.0 ± 0.04 to 3.6 ± 0.4 normalized gMFI]; *p* = 0.0121) on IgG1-mediated respiratory burst was found (Figure 5A). This was also true for phagocytosis, but to a lesser extent (1.3-fold [from 17.7 ± 3.7 to 22.4 ± 3.7 # erythrocytes per 100 PMNs]; *p* = 0.0249) (Figure 5B). The enhancing effect of CRP was mainly facilitated by FcγRIa, as the effect was strongly reduced or abolished upon FcγRIa blocking in any possible combination for respiratory burst (from +2.6 ± 0.5 to ≤ +0.3 ± 0.1 normalized gMFI; *p* = 0.0121 and *p* ≥ 0.0028) as well as phagocytosis (from +4.7 ± 1.3 to ≤ +0.6 ± 0.5 # erythrocytes per 100 PMNs; *p* = 0.0249 and *p* ≥ 0.0151) (Figures 5A,B). Here, blocking of FcγRII, FcγRIII, or both, negatively impacted the enhancing CRP effect, particularly for phagocytosis (≤ +1.7 ± 1.4 # erythrocytes per 100 PMNs; *p* ≥ 0.3113). FcαRI seemed to have a slight effect on the CRP enhancement for respiratory burst only (+1.7 ± 0.6 normalized gMFI; *p* = 0.0579), although not consistently when tested in combination with FcγRII/III-blocking (*p* ≤ 0.0414). Taken together, CRP-FcR interactions seem to be involved in the CRP enhancement of IgG-mediated PMN respiratory burst and phagocytosis.

IgG1-mediated cytotoxicity also strongly depended on FcγRIa (*p* < 0.0001), but was highly sensitive to blocking of FcγRII as well (*p* < 0.0001) (Figure 5C and Supplementary Figure 11). Notably, FcγRIII blocking did not affect baseline cytotoxicity (*p* = 0.9990), except in the absence of FcγRIa which seemed to partially restore the cytotoxic response. This is in agreement with the recently reported decoy function of FcγRIIIb on FcγRIIa-mediated tumor cell trogocytosis and cytotoxicity by PMNs (52). However, also in agreement with that work, we found that blocking FcγRIIIb could negatively affect phagocytosis (Figure 5B). As expected, blocking of FcαRI did not affect IgG1-mediated cytotoxicity (*p* = 0.8629). The CRP enhancement of cytotoxicity (from 30.0 ± 1.4 to 49.1 ± 1.7%; *p* = 0.0061) was intact after blocking of all low-affinity FcγRs as well as FcαRI in any combination (≥ +14.9 ± 3.5; *p* ≤ 0.0240). However, after FcγRIa blocking the CRP-mediated amplification of cytotoxicity was absent (≤ +0.7 ± 0.6; *p* ≥ 0.1403) or even slightly negative (−3.1 ± 0.4; *p* = 0.0034).

### CRP Enhancement of IgG2-Mediated Cytotoxicity Is Directed Through FcγRIa

The results above might suggest that FcγRIa-CRP interactions are crucial in enhancement of PMN cytotoxicity. However, an important limitation of this IgG1-based setup is the overlap of IgG1 and CRP in FcγR binding, i.e., FcγRIa and FcγRII blocking already markedly reduced cytotoxicity in the absence of CRP (Figure 5C). To gain more insight into the potential role of CRP-FcγRIa interactions and to circumvent FcγRIa-binding overlap by IgG1 and CRP, we switched to IgG2 as effector molecule as this subclass can only bind FcγRIIa, with no affinity to either FcγRIa or FcγRIIIa/b (61–63). However, as IgG2 only binds FcγRIIa (H131> R131), it has less potential to form a stable immunological synapse, that is further impeded by negative electrostatic charge provided by the cellular glycocalyx (49). Indeed, IgG2 only gave proper ADCC responses after trimming the erythrocyte glycocalyx with protease bromelain and using activated PMNs from individuals expressing the higher affinity FcγRIIa^H/H131^ phenotype (Supplementary Figure 15). Similar to our results with IgG1 (Figure 3L), CRP enhanced IgG2-mediated cytotoxicity, albeit with even stronger relative enhancement (236.1 ± 38.4%; *p* < 0.0001) (Supplementary Figure 15). Using IgG1 consistently showed lower CRP-mediated enhancement of baseline cytotoxicity, also after bromelain treatment of erythrocytes (mean relative enhancement by CRP 2.1 ± 0.4-fold higher using IgG2 than IgG1) (Supplementary Figure 16). As expected, FcγRII blocking in any combination resulted in complete absence of the IgG2-mediated cytotoxicity response (from 17.0 ± 1.6 to ≤ 0.2 ± 0.05%; *p* ≤ 0.0003), whilst FcγRIa, FcγRIII and FcαRI blocking did not have any impact (*p* ≥ 0.9730) (Figure 5D), confirming that IgG2 functions exclusively through FcγRIIa. However, blocking FcγRIa abrogated the CRP-mediated enhancement of cytotoxicity (from +42.8 ± 3.4 to ≤ +1.9 ± 1.6%; *p* = 0.0011 and *p* ≥ 0.2315) without affecting baseline effector function of IgG2 through FcγRIIa (*p* ≥ 0.9730), indicating the main amplification of IgG-mediated cytotoxicity by CRP is through FcγRIa. These IgG2-based setups did not lead to any respiratory burst or phagocytosis which seemed to depend largely on IgG interactions with FcγRIa and to a lesser extent FcγRIIIb (Figures 5A,B and Supplementary Figure 4).

### CRP Enhances IgG1-Mediated Tumor Cell Killing by Activated PMNs

**Figure 6:**
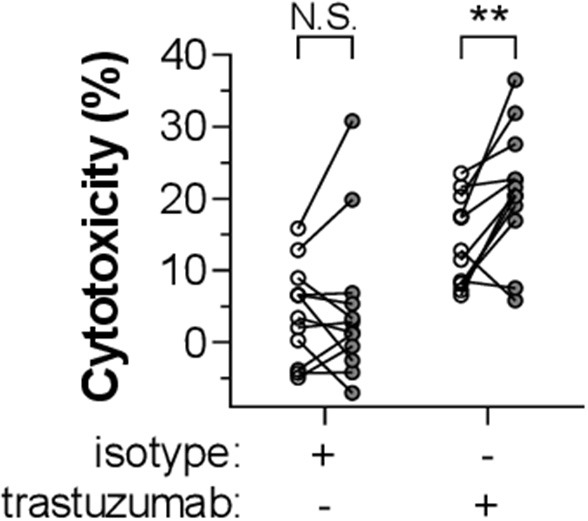
CRP enhances IgG1-mediated tumor cell killing by activated PMNs. The role of CRP in enhancing IgG1-mediated cytotoxicity by G-CSF/IFN-γ-stimulated PMNs toward a breast cancer tumor cell line (SKBR3) was determined by adding CRP (10 μg/ml) to the reaction (gray symbols; or 0 μg/ml, depicted as white symbols) in the presence of 10 μg/ml trastuzumab or isotype control. Cytotoxicity was measured as the percentage of lysed target SKBR3 cells (%). This experiment has been performed in seven independent experiments using PMNs from 12 donors. Data points (circles) each represent the mean of triplo values and are paired for each specific donor. Significant differences between conditions with or without CRP have been determined using two-tailed paired *t-*test. **p < 0.01; N.S. not significant.

Finally, we investigated whether CRP can act as a general enhancer of IgG-mediated cellular destruction, e.g., in mAb-mediated tumor responses. Activated PMNs are known to induce killing of SKBR3 human breast cancer cells through anti-Her2 IgG1 (trastuzumab/Herceptin) as opsonizing antibody (52). Our results demonstrate that CRP (10 μg/ml) indeed significantly amplifies the lysis of trastuzumab-opsonized SKBR3 cells by activated PMNs with a mean relative enhancement of 73.4 ± 23.9% (*p* = 0.0047) (Figure 6).

## Discussion

**Figure 7:**
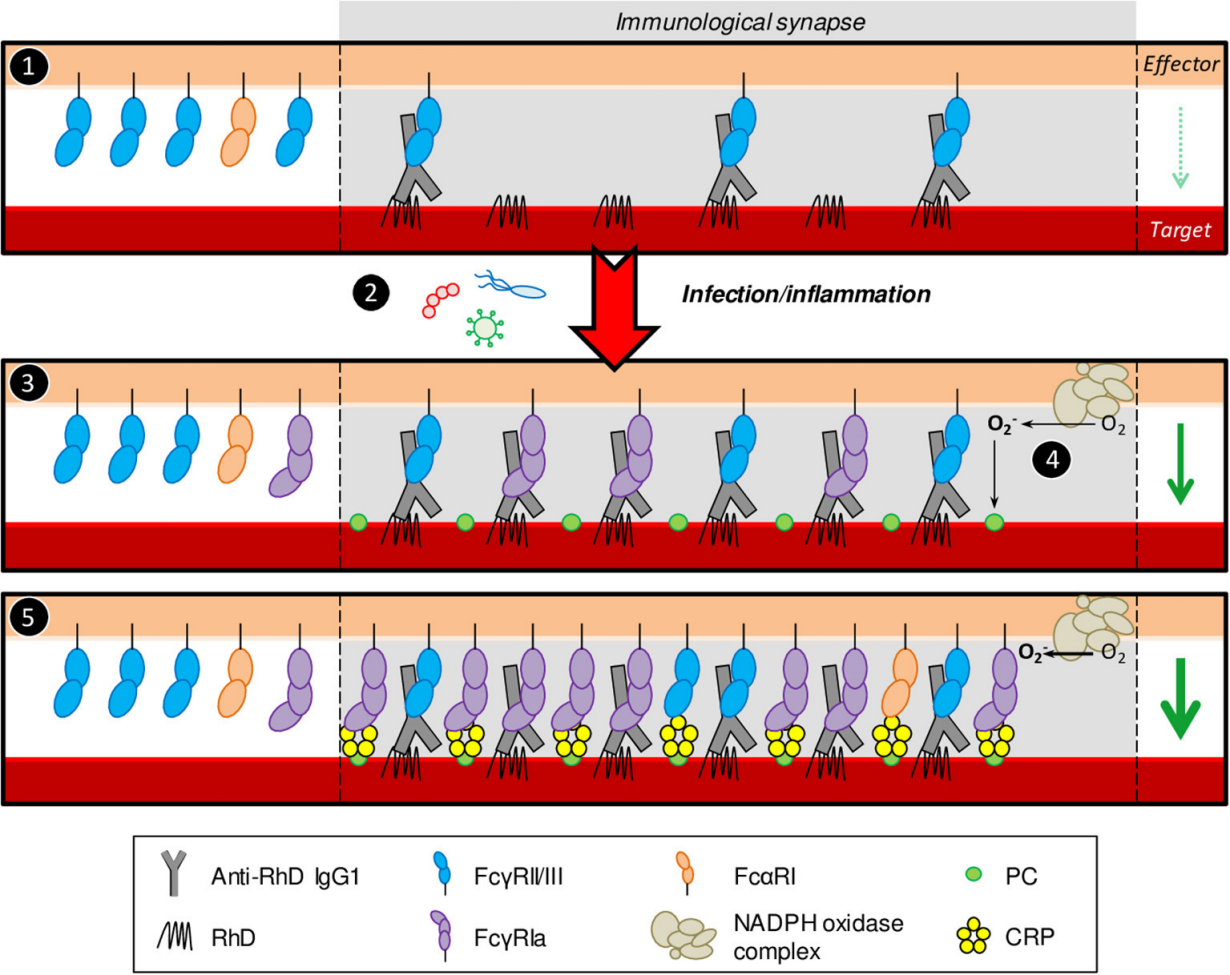
Proposed model for CRP enhancement of IgG-mediated erythrocyte destruction. **(1)** During the pre-symptomatic phase autoantibody levels are low resulting in low target cell opsonization. Consequently, immunological synapses are not stable enough to trigger potent effector functions by naïve PMNs. **(2)** Infection by pathogens inflicts inflammatory conditions and acute-phase responses which are known to cause upregulation of serum CRP levels through pro-inflammatory cytokines. **(3)** Next to CRP upregulation, these conditions also induce FcγRIa expression on activated PMNs. High-affinity FcγRIa-IgG interactions stabilize the synapse through more receptor clustering, inflicting potent effector functions toward the target cell, including, but not limited to, respiratory burst. **(4)** The PMN NADPH oxidase machinery converts O_2_ to ROS (${\text{O}}_{2}^{-}$) which oxidizes phosphatidylcholine residues on the target cell plasma membrane leading to exposure of PC head groups. **(5)** CRP binds exposed PC and interacts with PMN FcγRs (and potentially also FcαRI), recruiting more receptors to the synapse, causing more clustering resulting in even more potent effector functions. Taken together, infection/inflammatory conditions make PMNs more sensitive for low level autoantibodies and CRP, thereby decreasing the threshold for target destruction. PMN effector functions (respiratory burst, phagocytosis, cellular cytotoxicity) are indicated by the green arrow on the right side in each panel where the arrow width corresponds to response magnitude. The relative abundance of different FcR types outside the immunological synapse is schematic and does not completely reflect reality. Generally, naïve PMNs have been reported to express <2,000 copies of FcγRIa, ~20,000 copies of FcγRIIa, 100,000–300,000 copies of FcγRIIIb, and around 10,000 copies of FcαRI per cell (58). IFN-γ/G-CSF stimulation induces FcγRIa expression 10-times and leads to up to 50% shedding of FcγRIIIb (52, 56). FcγRIIa levels are unaffected or subtly lower after IFN-γ/G-CSF stimulation (56) and FcαRI expression is not affected at all (57).

Here, we demonstrate, for the first time, CRP's ability to enhance IgG-mediated phagocytic and cytotoxic responses toward opsonized erythrocytes and tumor cells through FcγRs. This suggests its potential to aggravate pathologies where erythrocytes are targeted by antibodies and to increase effectivity of anti-tumor antibodies. Importantly, relatively low-level CRP already amplified effector functions of myeloid cells expressing considerable FcγRIa, especially activated PMNs. This is in favor of the proposed pathological role of elevated low-level (high-sensitivity) CRP in thrombocytopenia and cardiovascular disease (4, 6, 64). During inflammation and infection both CRP expression and PMN activation level are upregulated, which might explain sudden relapses or exacerbations of antibody-mediated autoimmune diseases following infection. We hypothesize that prior to infection autoantibodies cause mild symptoms, but during acute-phase CRP upregulation causes disease relapse by amplifying IgG-mediated cell destruction. In parallel, induced FcγRIa expression makes activated PMNs more sensitive for low-level autoantibodies and CRP, thereby decreasing the threshold for target destruction (summarized in Figure 7).

We found that CRP-mediated exacerbation of phagocytosis depends on the presence of calcium, consistent with CRP's calcium-dependent binding to PC. This is also analogous to our previous work that showed calcium dependency of CRP-mediated enhancement of PMN effector functions toward IgG1-opsonized thrombocytes (4). This preceding study also demonstrated that phagocyte NADPH oxidase activity caused PC exposure, enabled CRP binding to thrombocytes and was required for CRP-mediated enhancement of effector functions (4). We confirmed that similar mechanisms apply for IgG-mediated erythrocyte destruction, as inhibition of NADPH oxidation abolished CRP-mediated enhancement of phagocytosis without affecting ADCP.

It has long been a matter of debate which specific FcγR types bind CRP and this was not fully documented prior to this study. Therefore, we quantified CRP binding to all human FcγRs and FcαRI side-by-side using an antibody- and label-free SPR platform. We found that CRP binds all FcRs with low affinity. Consistent with our findings is the specific binding of CRP and CRP-opsonized PC-labeled erythrocytes to cells expressing FcγRIa (23, 33) or FcγRIIa (23, 34, 35) and SPR data where CRP bound immobilized FcγRIa (22, 23), FcγRIIa/b, FcγRIIIa, and FcαRI (22, 36) with affinities in the μM range. In addition, we demonstrated that FcγR polymorphisms, which can dramatically impact IgG binding, do not seem to affect CRP binding strength.

CRP exclusively enhanced effector functions of FcγRIa-expressing cells (e.g., monocytes and activated PMNs, but not naïve PMNs or NK cells) which were effectively blocked by anti-FcγRIa antibodies. Despite similar binding strength of CRP to FcγRIIa/b, FcγRIIIa/b, and FcαRI, these receptors seemed to be functionally less involved in the CRP-mediated enhancement. A possible explanation might be more efficient avidity effects of CRP toward FcγRIa than to other FcγRs. In addition, affinities were not independent from R_max_ suggesting that higher cellular FcγR density/clustering may markedly enhance CRP binding efficacy. This also implies that our reported low-affinity K_D_ values (at R_max_ = 500) might be an underestimation and not necessarily representative for certain native situations, e.g., immunological synapse. Another interesting thought is that FcγRIa bridges bigger distances and/or alters stoichiometry between effector and target cells through its unique third extracellular domain (D3), a potential spacer between the plasma membrane (PM) and D2-D1 (38), and thereby inflicting more optimal docking into opsonizing CRP contributing to synapse stabilization.

Notably, one limitation in this study is that the anti-FcγR agents used, known to block IgG binding, were assumed to also block CRP binding to these receptors. This assumption is based on the competition between CRP and IgG for FcγR binding, their overlapping binding sites on the FcγR D2 domain (14, 22, 23, 38), and the homology between IgG and CRP-motifs involved in FcγR binding (39). Whereas, the blockers of FcγRIa (clone 10.1) and FcαRI (MIP7C) have been verified previously to also efficiently block CRP binding (26, 33, 37, 65), the anti-FcγRII/III fragments (clones 7.3 and 3G8, respectively) are less well-characterized for blocking of CRP-FcγR interactions, which should be taken into consideration interpreting the data. However, several blocking conditions involving these agents do seem to subtly affect CRP specific effects, suggesting (at least partial) blocking of CRP binding to FcγRII/III.

We and others (4, 6) demonstrated that CRP was, as expected, inert toward healthy target cells. Only after IgG opsonization, CRP exerted additional FcR-mediated effector functions. We therefore hypothesize (Figure 7) that opsonizing IgG first needs to establish immunological contact with effector cells through FcγRs, triggering respiratory burst responses. Subsequently, IgG-opsonized target PM will be oxidized leading to PC exposure on the cell surface, allowing for calcium-dependent CRP binding. After opsonization, CRP's A-face can bind and recruit additional FcRs (mainly FcγRIa) to the IgG-based immunological synapse causing more FcR clustering and thereby enhancing effector functions and subsequent target destruction. This effect was particularly clear using IgG2, where CRP-mediated enhancement was exceptionally strong, probably since IgG2, unlike IgG1, does not bind FcγRIa and cannot compete with CRP for FcγRIa binding and recruitment. With that in mind, it is particularly interesting that CRP was initially found as an opsonin to C-polysaccharide (PC-rich teichoic acid of *Streptococcus pneumoniae*) to which IgG2 responses dominate (66). In addition, CRP levels upon pneumococcal or other Gram-positive infections are known to be increased efficiently (13).

Anti-RhD IgG1-mediated monocyte and PMN cytotoxicity appeared to be directed through FcγRIa and FcγRIIa (whilst IgG1-mediated phagocytosis was facilitated through FcγRIa and to a lesser extent FcγRIIIb, but not FcγRIIa), with monocytes constitutively expressing more FcγRIa than activated PMNs. Remarkably, CRP-mediated enhancement of erythrocyte lysis was only consistent for activated PMNs. An explanation for this is that monocytes are less potent in exerting efficient respiratory burst responses (67) which is potentially required for conversion of membrane phosphatidylcholine to PC-exposing lysophosphatidylcholine providing CRP binding platforms (68). Consistent with this was our observation that PMNs triggered more efficient IgG-dependent NADPH oxidase activity than monocytes in the presence of CRP.

Besides elevated CRP levels in infectious diseases after acute-phase responses, elevated CRP is also found in auto- or alloimmune diseases, e.g., ITP (4, 6), FNAIT (4) and TRALI (9). In these pathologies the underlying inflammation, including CRP levels, often reflects disease severity. Besides some case studies, CRP levels have never been systematically studied in AIHA or HDFN patients. Especially AIHA patients are likely to have episodes of elevated CRP levels and FcγRIa expression since AIHA is often secondary to a broad range of inflammatory conditions and IL-33 levels are reportedly elevated (41, 42). Moreover, autoimmune diseases are generally known to initiate, exacerbate or relapse upon bacterial or viral infections which inflict CRP upregulation. The potency of PMNs to efficiently eliminate erythrocytes in AIHA is in agreement with recent findings (40) and case studies reporting PMN-erythrocyte rosettes in blood smears from AIHA patients (46, 69–72).

Interestingly, CRP is also elevated in malaria (73) where erythrocytes can be lysed through antibodies targeting parasite antigens displayed on infected cells and/or phosphatidylserine on uninfected cells (74, 75). Since CRP levels correlate with disease severity (73), CRP may also directly participate in exacerbation of antibody-mediated erythrocyte destruction next to its demonstrated functional involvement in complement-mediated erythrocyte destruction in malaria (76, 77).

Since CRP apparently potentiates IgG-mediated destruction of several types of target cells, its application in immunotherapies (cancer and infectious diseases) can be envisioned. Conversely, patients suffering from IgG-mediated cytolysis may benefit from treatment of underlying infections and therapeutic approaches modulating CRP levels (e.g., 1,6-bis[PC]-hexane) (78). Importantly, before such therapeutic applications, CRP's mode of action should first be further dissected. Based on our findings it would, for example, be relevant to investigate which signaling cascades CRP triggers and if it affects cytokine production in activated PMNs, likewise recently was performed for macrophages (25).

In conclusion, we provide the first evidence for CRP as an enhancer of *in vitro* IgG-mediated erythrocyte and tumor cell destruction. CRP appeared to function through FcγRs, with an essential role for FcγRIa. Our data are consistent with and extent current knowledge on CRP's role in antibody-mediated cell destruction. CRP's precise working mechanism should be further dissected, as well as its *in vivo* effects and prognostic value in IgG-mediated hemolytic pathologies. In addition, CRP should also be further evaluated as a potential therapeutic adjuvant for tumor eradication.

## Data Availability Statement

The original contributions presented in the study are included in the article/Supplementary Materials, further inquiries can be directed to the corresponding author/s.

## Ethics Statement

Ethical review and approval was not required for the study on human participants in accordance with the local legislation and institutional requirements. The patients/participants provided their written informed consent to participate in this study.

## Author Contributions

AT performed and designed all erythrocyte cytotoxicity experiments. MT performed and designed phagocytosis and respiratory burst experiments which were supervised by AT. AT and MT isolated effector cells and prepared target cells. AT and AB performed and designed SPR binding experiments. Binding affinity values were calculated by AB. LT and HF designed and performed tumor cell cytotoxicity experiments which were supervised by TB. ST cloned and produced anti-RhD IgG1 and glyco-engineered afucosylated anti-RhD IgG1. ST cloned and AT produced anti-RhD IgG2. TK and SN generated and provided *FCGR2A*-H/R131 (p.His166Arg) genotyping information. GB, JM, and WE performed site-specific FcR biotinylation. TR and CS gave critical input on experimental approach. GV supervised the project and designed experiments. AT and GV wrote the manuscript which was approved and critically reviewed by all co-authors. All authors contributed to the article and approved the submitted version.

## Conflict of Interest

The authors declare that the research was conducted in the absence of any commercial or financial relationships that could be construed as a potential conflict of interest.
