# Supplementary material for: C-Reactive Protein Enhances IgG-Mediated Cellular Destruction Through IgG-Fc Receptors in vitro
Source: Front Immunol. 2021 Mar 15;12:594773. doi: 10.3389/fimmu.2021.594773 (PMC8006934; doi:10.3389/fimmu.2021.594773)
Supplement: Supplementary file 1 [file Data_Sheet_1.pdf]

Supplementary material

C-reactive protein enhances IgG-mediated cellular destruction through IgG-Fc receptors *in vitro*

A. Robin Temming[1], Matthias Tammes Buirs[1], Arthur E. H. Bentlage[1], Louise W. Treffers[2], Hannah Feringa[2], Steven W. de Taeye[1,3], Taco W. Kuijpers[2,4], Sietse Q. Nagelkerke[2,4], Giso Brasser[5], Juk Yee Mok[5], Wim J.E. van Esch[5], Timo K. van den Berg[2], Theo Rispens[3], C. Ellen van der Schoot[1] and Gestur Vidarsson[1]

[1] *Department of Experimental Immunohematology, Sanquin Research and Landsteiner Laboratory, Amsterdam University Medical Center, University of Amsterdam, 1066 CX Amsterdam, the Netherlands*
[2] *Department of Blood Cell Research, Sanquin Research and Landsteiner Laboratory, Amsterdam University Medical Center, University of Amsterdam, 1066 CX Amsterdam, the Netherlands*
[3] *Department of Immunopathology, Sanquin Research and Landsteiner Laboratory, Amsterdam University Medical Center, University of Amsterdam, 1066 CX Amsterdam, the Netherlands*
[4] *Department of Pediatric Immunology, Rheumatology and Infectious diseases, Emma Children's Hospital, Amsterdam University Medical Center, University of Amsterdam, 1105 AZ Amsterdam, the Netherlands*
[5] *Sanquin Reagents, Sanquin, 1066 CX Amsterdam, the Netherlands*

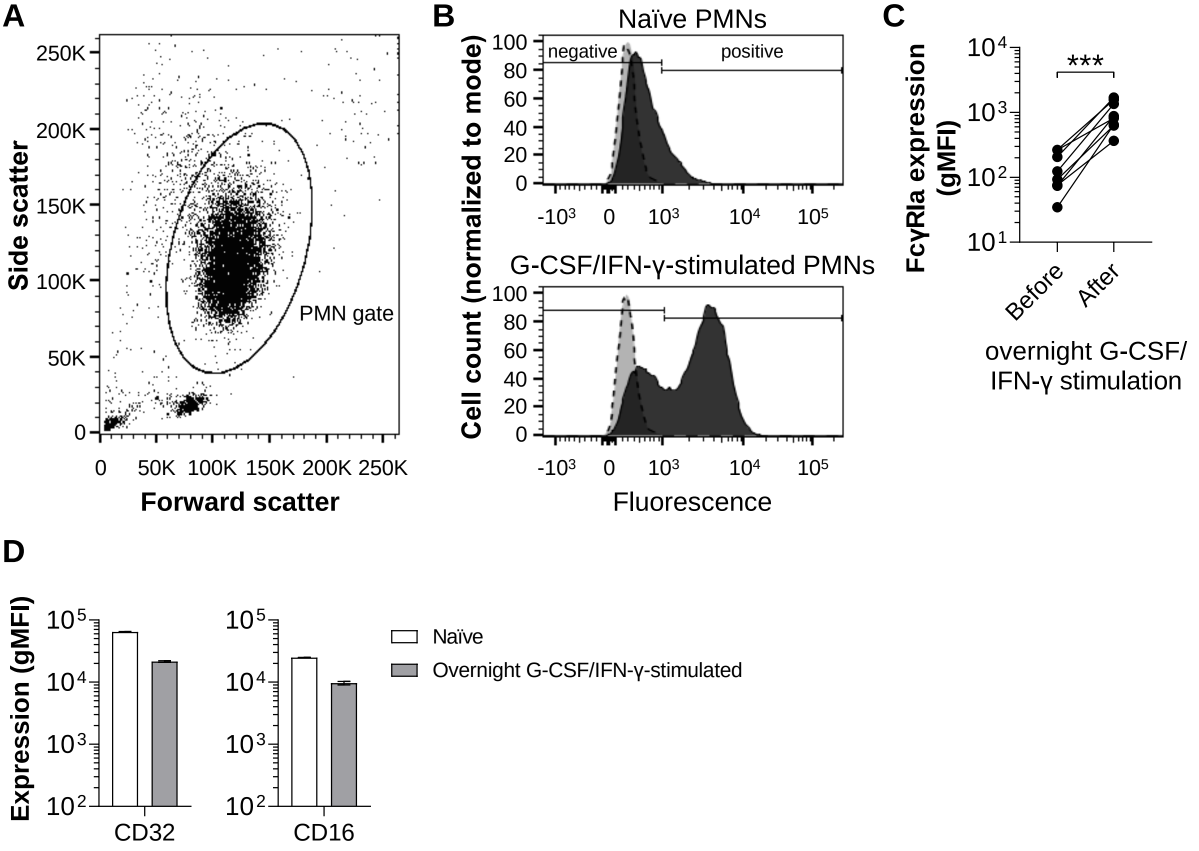

SUPPLEMENTAL FIGURE 1: FcγRIa expression on naïve and G-CSF/IFN-γ-stimulated PMNs. **(A)** Gating strategy of the PMN fraction based on their characteristic forward and sideward scatter pattern. **(B)** Representative flow cytometry histograms depicting binding of biotinylated anti-CD64 mAb (black histograms) to naïve (upper panel) and overnight (O/N) G-CSF/IFN-γ-stimulated PMNs (lower panel). For negative control purposes unstained PMNs (dashed histograms) and detection control (fluorescently-labeled streptavidin only; grey histograms) were included. Threshold for positivity is also indicated in each panel and was set based on the unstained PMN signals. **(C)** FcγRIa expression levels (gMFI) on PMNs before and after O/N incubation with 10 ng/ml G-CSF and 50 ng/ml IFN-γ. Data points represent mean values of duplo data and PMNs from the same donor are connected with a line. **(D)** Representative expression patterns (gMFI ± S.E.M.) of FcγRII (CD32, left graph) and FcγRIII (CD16, right graph) on PMNs before (white bars) and after (grey bars) O/N incubation with 10 ng/ml G-CSF and 50 ng/ml IFN-γ. Two-tailed paired t-test was used to determine significant differences. *** $p < 0.001$.

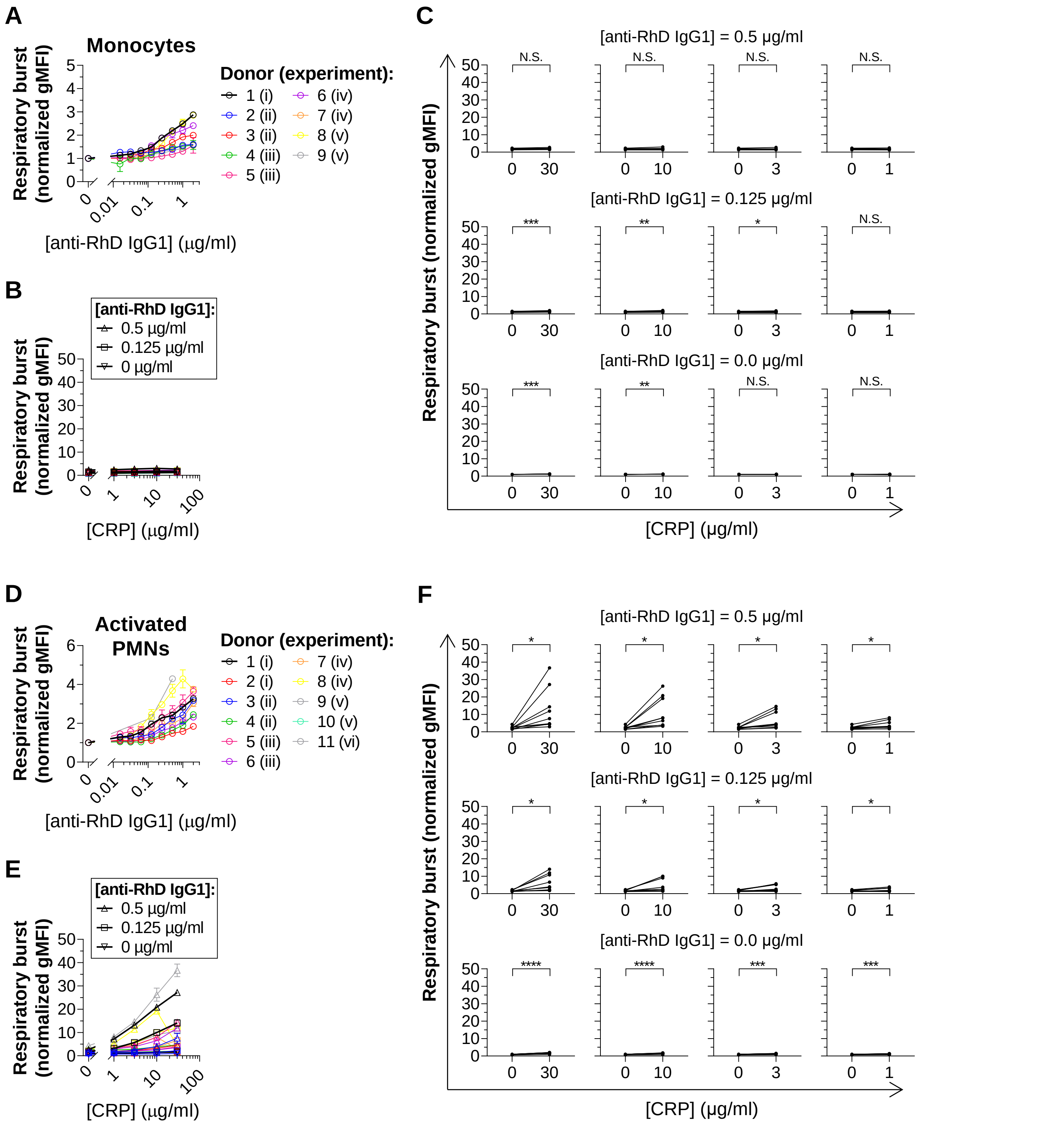

SUPPLEMENTAL FIGURE 2: CRP effect on IgG1-mediated respiratory burst activity of monocytes and activated PMNs towards opsonized erythrocytes. All replicates for IgG1 concentration-dependent respiratory burst activity (normalized gMFI ± standard error of the mean [S.E.M.]; signals of the 0 μg/ml IgG1 condition were set to 1.0) of monocytes (**A**; 9 donors, 5 independent experiments) and G-CSF/IFN-γ-stimulated PMNs (**D**; 11 donors, 6 independent experiments) towards pre-opsonized target erythrocytes. **(B, E)** Titration curves of CRP dilutions (30, 10, 3, 1, 0 μg/ml) added to the reaction with different anti-RhD IgG1 backgrounds (0.5, 0.125 and 0 μg/ml) using effector cells from the same donors as in **A, D**. **(C, F)** The impact of CRP addition (30, 10, 3, 1 μg/ml) on respiratory burst activity triggered by 0, 0.125 and 0.5 μg/ml anti-RhD IgG1 using effector cells from different donors. All individual data points represent duplo data. Two-tailed paired t-test was used to determine significant differences. * $p \leq 0.05$; ** $p < 0.01$; *** $p < 0.001$; **** $p < 0.0001$; N.S. not significant.

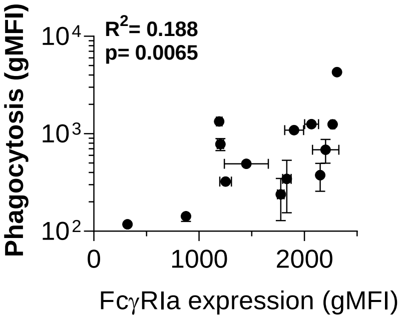

SUPPLEMENTAL FIGURE 3: PMN FcγRIa expression correlates with magnitude of phagocytosis. PMN FcγRIa expression levels (gMFI ± S.E.M.) and the corresponding phagocytosis (gMFI ± S.E.M.) of erythrocytes opsonized with 0.125 µg/ml anti-RhD IgG1 were determined by flow cytometry. Correlation between FcγRIa expression and phagocytosis was determined by linear regression. Each data point represents mean phagocytosis (duplo data) performed by one specific PMN donor at the corresponding mean FcγRIa expression (duplo data).

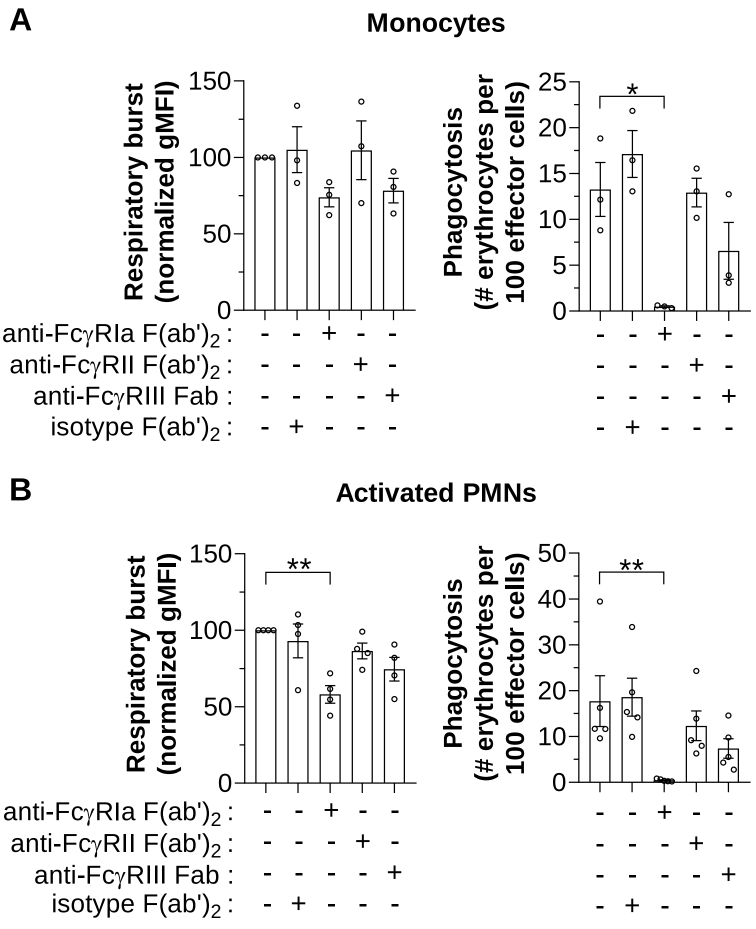

SUPPLEMENTAL FIGURE 4: Role of FcγRs in IgG1-mediated respiratory burst and phagocytosis by monocytes and activated PMNs. The involvement of individual Fc**γ**R types in IgG1-mediated monocyte **(A)** and G-CSF/IFN-γ-stimulated PMN **(B)** respiratory burst activity (left panels) and phagocytosis (right panels) to pre-opsonized (0.125 µg/ml anti-RhD IgG1) erythrocytes was determined using a panel of Fc**γ**R blocking agents. For blocking, effector cells were pre-incubated with the respective blocking agent, or isotype equivalent, at a concentration of 10 µg/ml. For respiratory burst data, gMFI values from conditions without blocking were normalized to 1.0 and phagocytosis is depicted as phagocytic index (# erythrocytes per 100 effector cells). Bar graphs indicate the mean value ± S.E.M. of data points (circles) each representing the mean of duplo values of one specific donor (3 monocyte donors; 4-5 PMN donors). Significant differences between blocking conditions have been determined using one-way ANOVA. * $p \leq 0.05$; ** $p < 0.01$.

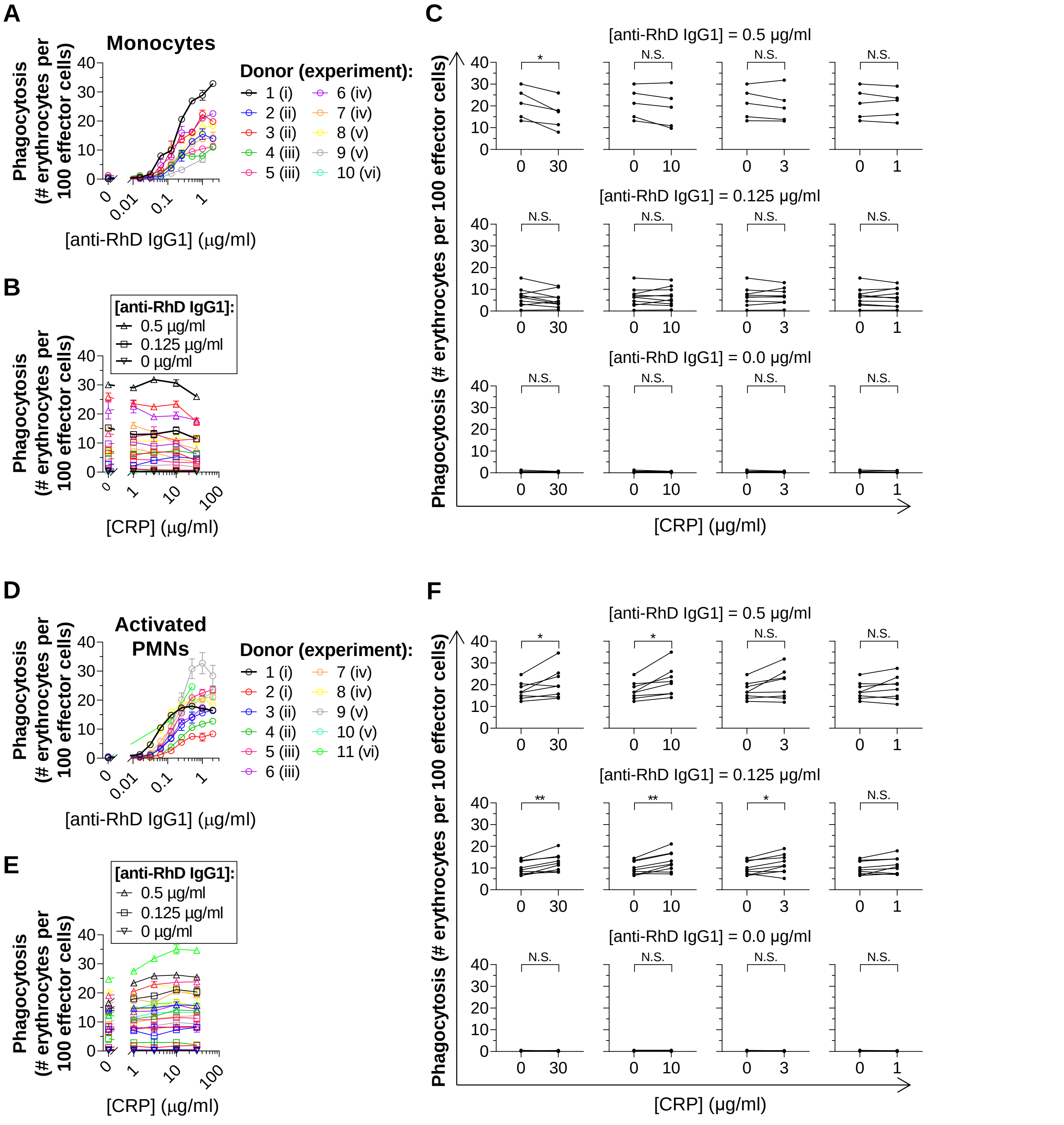

SUPPLEMENTAL FIGURE 5: CRP effect on IgG1-mediated erythrophagocytosis by monocytes and activated PMNs. All replicates for IgG1 concentration-dependent phagocytosis (phagocytic index as number [#] of erythrocytes per 100 effector cells ± S.E.M.) of monocytes (**A**; 10 donors, 6 independent experiments) and G-CSF/IFN-γ-stimulated PMNs (**D**; 11 donors, 6 independent experiments) towards pre-opsonized target erythrocytes. **(B, E)** Titration curves of CRP dilutions (30, 10, 3, 1, 0 μg/ml) added to the phagocytosis reaction with different anti-RhD IgG1 backgrounds (0.5, 0.125 and 0 μg/ml) using effector cells from the same donors as in **A, D**. **(C, F)** The impact of CRP addition (30, 10, 3, 1 μg/ml) on phagocytosis triggered by 0, 0.125 and 0.5 μg/ml anti-RhD IgG1 using effector cells from different donors. All individual data points represent duplo data. Two-tailed paired t-test was used to determine significant differences. * $p \leq 0.05$; ** $p < 0.01$; N.S. not significant.

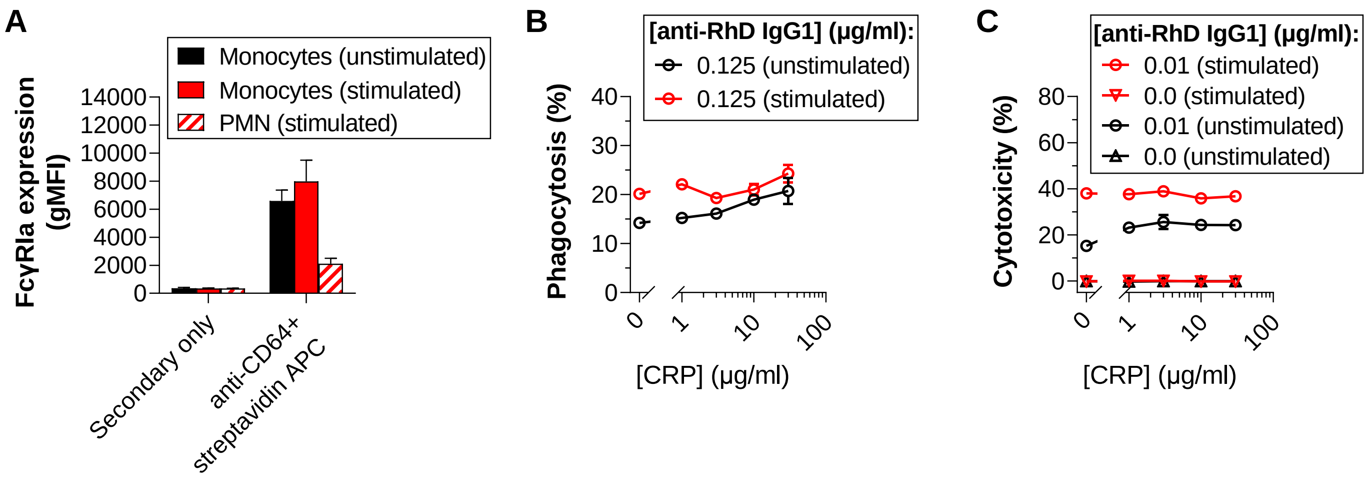

SUPPLEMENTAL FIGURE 6: (A) FcγRIa expression levels (gMFI + S.E.M.) on monocytes (2 donors) before (black bars) and after (red bars) O/N incubation with 10 ng/ml G-CSF and 50 ng/ml IFN-γ. To show the relatively high constitutive FcγRIa levels on unstimulated monocytes, FcγRIa expression levels on O/N G-CSF/IFN-γ-stimulated PMNs (2 donors) were included (dashed bars). **(B)** Phagocytosis responses (% positive cells ± S.E.M.) of unstimulated (black) and O/N G-CSF/IFN-γ-stimulated (red) monocytes towards anti-RhD IgG1 (0.125 µg/ml)-opsonized erythrocytes in the absence (0 µg/ml) or presence (1-30 µg/ml) of CRP. **(C)** Cytotoxic responses (% lysed target cells ± S.E.M.) of unstimulated (black) and O/N G-CSF/IFN-γ-stimulated (red) monocytes towards anti-RhD IgG1 (0.01 µg/ml)-opsonized or unopsonized (0.0 µg/ml) erythrocytes in the absence (0 µg/ml) or presence (1-30 µg/ml) of CRP. Data represent mean values of duplo (A) or triplo (B,C) data.

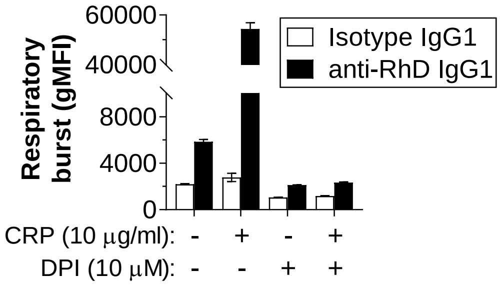

SUPPLEMENTAL FIGURE 7: IgG1-mediated respiratory burst activity by activated PMNs is enhanced by CRP and abolished by inhibiting the NADPH oxidase machinery. The dependency of the respiratory burst response (gMFI ± S.E.M.) on NADPH oxidase activity was studied by adding oxidation inhibitor DPI (10 µM) to the reaction. Target erythrocytes were pre-opsonized with 0.125 µg/ml anti-RhD IgG1 (black bars) or isotype control (white bars) and incubated with G-CSF/IFN-γ-stimulated PMNs in the presence or absence of 10 µg/ml CRP.

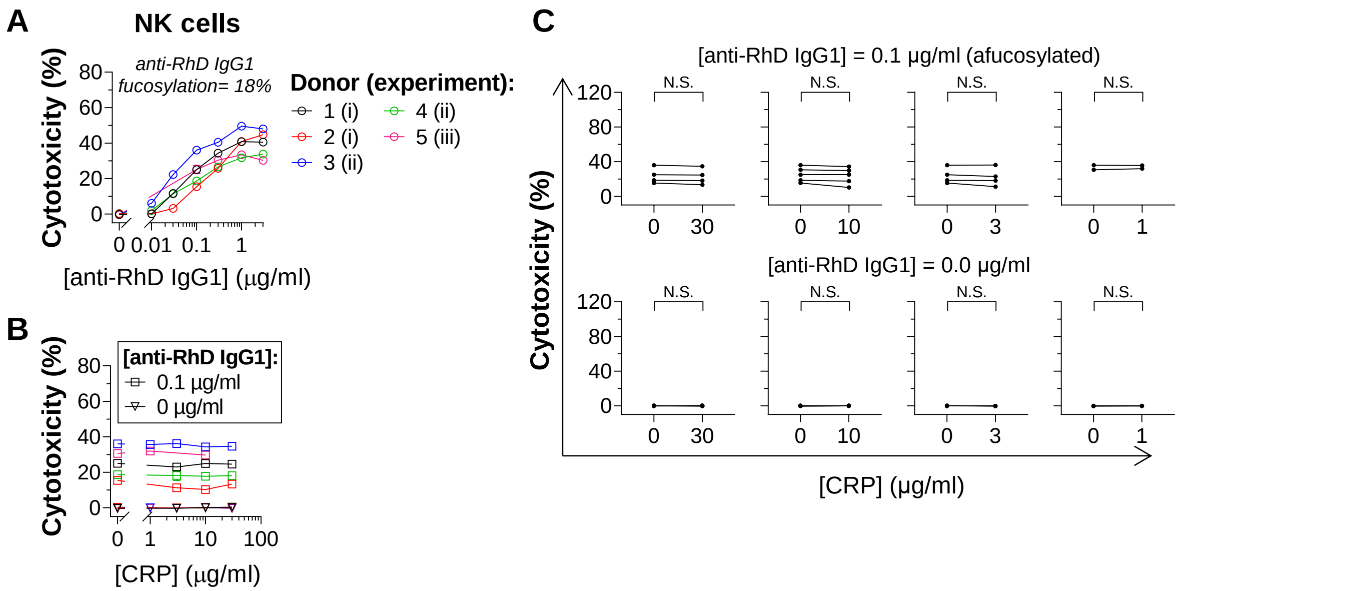

SUPPLEMENTAL FIGURE 8: CRP effect on NK cell cytotoxicity of opsonized erythrocytes using afucosylated IgG1. All replicates for IgG1 concentration-dependent cytotoxic responses (% lysed target cells ± S.E.M.) of NK cells (**A**; 5 donors, 3 independent experiments) towards opsonized target erythrocytes. **(B)** Titration curves of CRP dilutions (30, 10, 3, 1, 0 µg/ml) added to the cytotoxicity reaction with different afucosylated (18% vs. 94% for WT) anti-RhD IgG1 backgrounds (0.1 and 0 µg/ml) using effector cells from the same donors as in **A**. **(C)** The impact of CRP addition (30, 10, 3, 1 µg/ml) on cytotoxicity triggered by 0.1 and 0 µg/ml anti-RhD IgG1 using effector cells from different donors. All individual data points represent triplo data. Two-tailed paired t-test was used to determine significant differences. N.S. not significant.

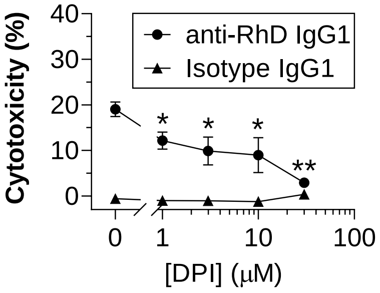

SUPPLEMENTAL FIGURE 9: IgG1-mediated cellular cytotoxicity by activated PMNs depends on respiratory burst activity. Titration curves of DPI dilutions (30, 10, 3, 1, 0 µg/ml) added to the cytotoxicity reaction in the presence of anti-RhD IgG1 (black circles; 0.01 µg/ml) or IgG1 isotype (triangle; 0.01 µg/ml) using effector cells. G-CSF/IFN-γ-stimulated PMNs were used as effector cells and data points represent triplo measurements of cytotoxicity (% of lysed target erythrocytes ± S.E.M.). Significant differences compared to the condition without DPI have been determined using one-way ANOVA. * $p \leq 0.05$; ** $p < 0.01$.

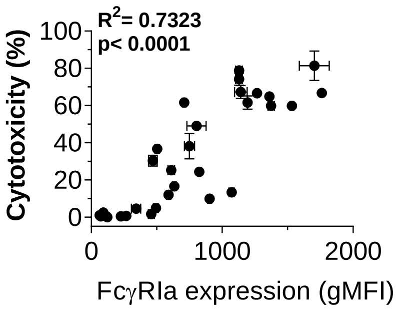

SUPPLEMENTAL FIGURE 10: PMN FcγRIa expression correlates with cellular magnitude of cytotoxicity. PMN FcγRIa expression levels (gMFI ± S.E.M.) were determined by flow cytometry. Corresponding cytotoxicity responses (% ± S.E.M.) depict the lysis of erythrocytes opsonized with 0.01 µg/ml anti-RhD IgG1. Correlation between FcγRIa expression and cytotoxicity was determined by linear regression. Each data point represents mean cytotoxicity (triplo data) performed by one specific PMN donor at the corresponding mean FcγRIa expression (duplo data).

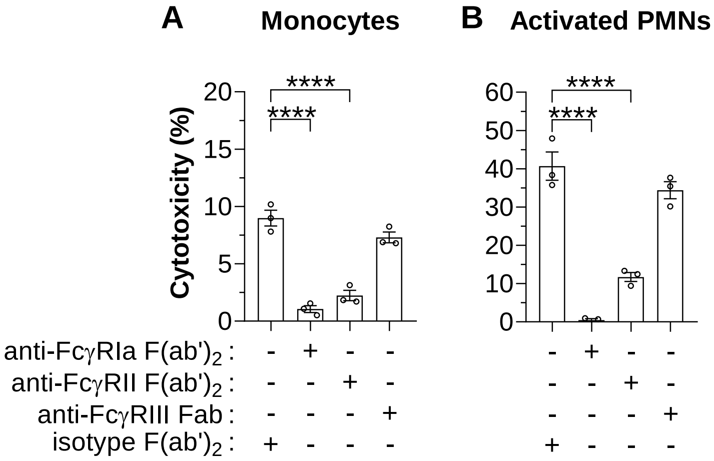

SUPPLEMENTAL FIGURE 11: Role of FcγRs in IgG1-mediated cellular cytotoxicity by monocytes and activated PMNs. The involvement of individual FcγR types in IgG1-mediated monocyte **(A)** and G-CSF/IFN-γ-stimulated PMN **(B)** cellular cytotoxicity (% of lysed target erythrocytes) to opsonized (0.01 µg/ml anti-RhD IgG1) erythrocytes was determined using a panel of FcγR blocking agents. For blocking, effector cells were pre-incubated with the respective blocking agent, or isotype equivalent, at a concentration of 10 µg/ml. Bar graphs indicate the mean value ± S.E.M. of data points (circles) each representing the mean of triplo values of one specific donor (3 monocyte donors; 3 PMN donors). Significant differences between blocking conditions have been determined using one-way ANOVA. **** $p < 0.0001$.

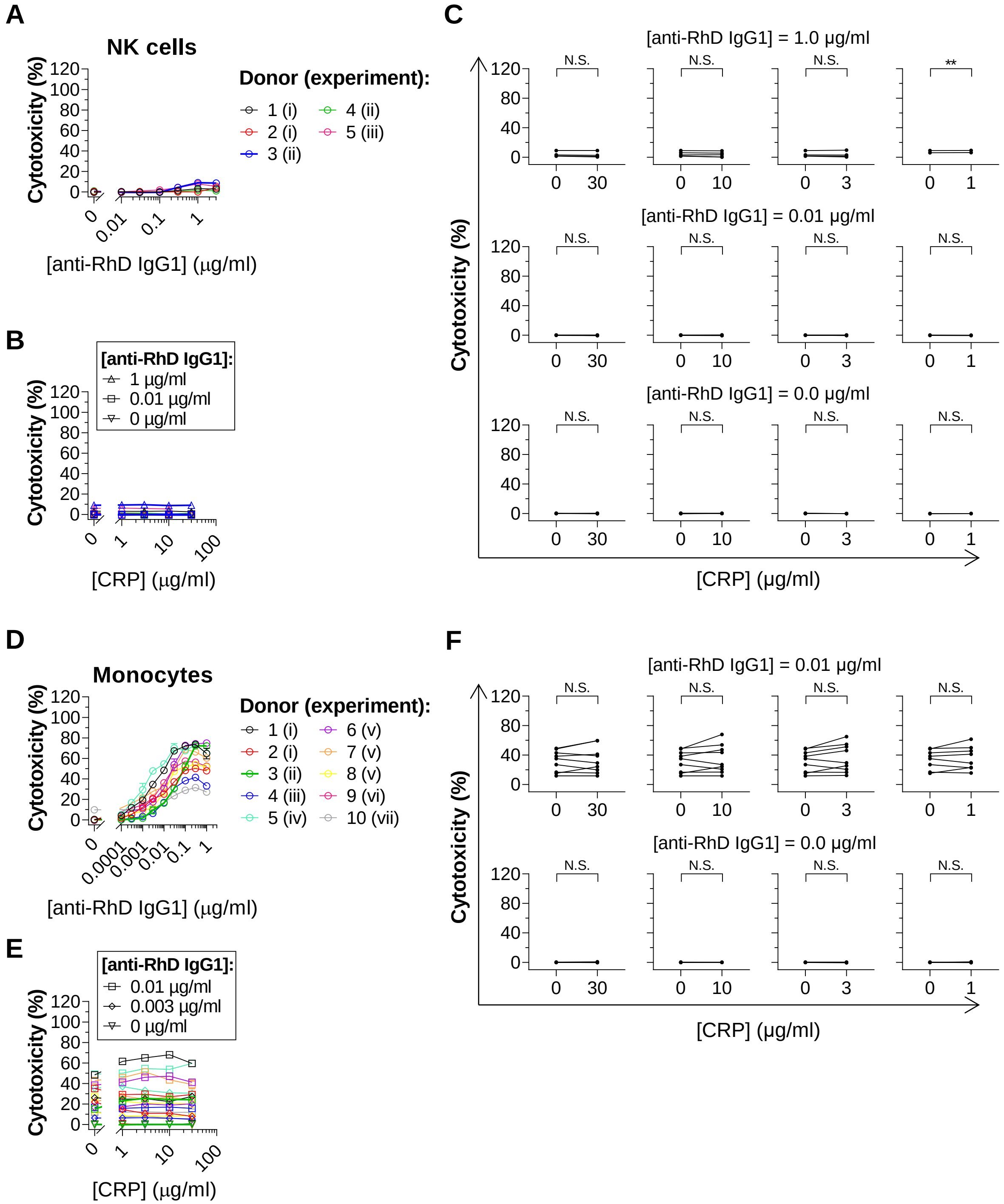

SUPPLEMENTAL FIGURE 12: Continued on the next page.

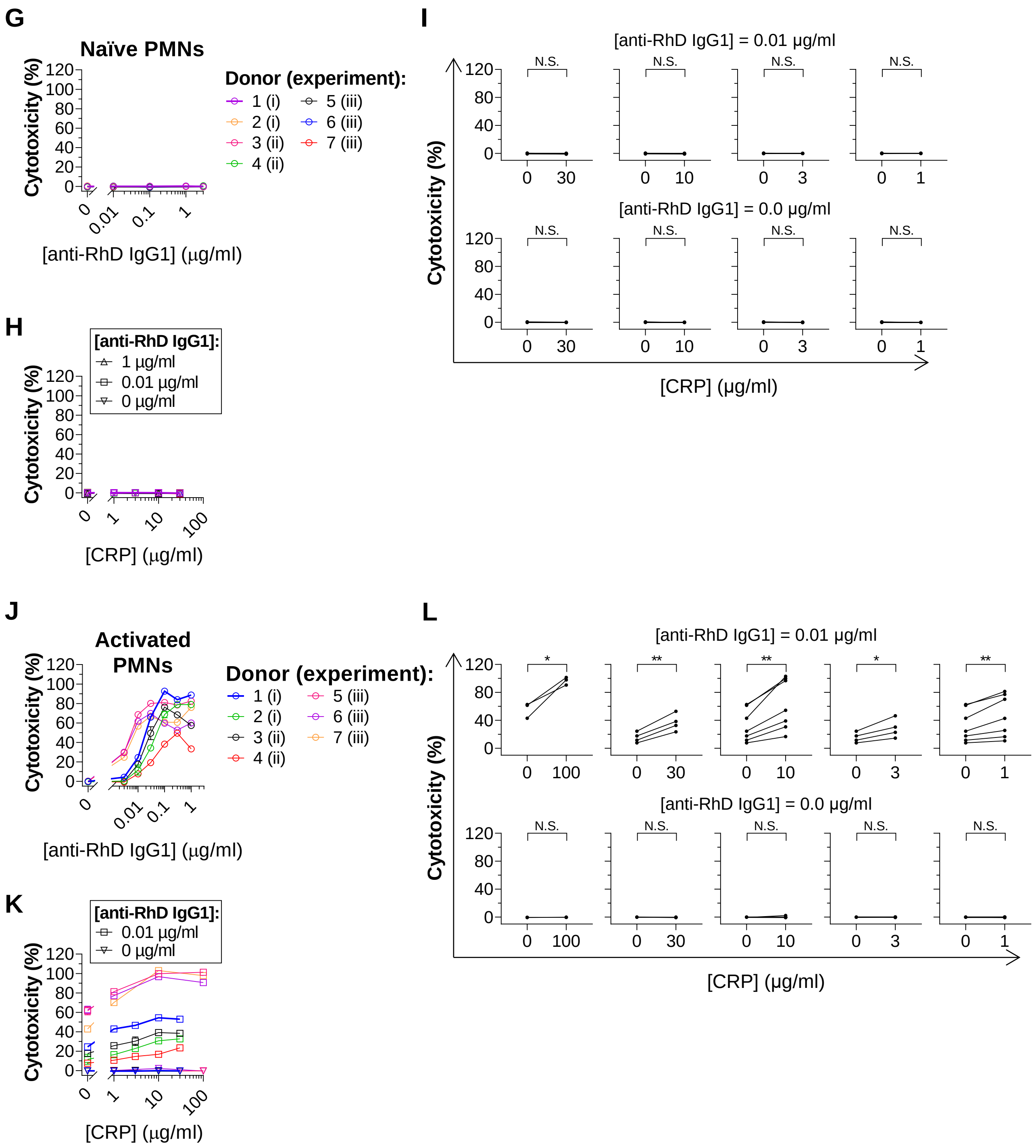

SUPPLEMENTAL FIGURE 12: CRP effect on IgG1-mediated cytotoxicity of opsonized erythrocytes by NK cells, monocytes, naïve PMNs and activated PMNs. All replicates for IgG1 concentration-dependent cytotoxic responses (% lysed target cells ± S.E.M.) of NK cells (**A**; 5 donors, 3 independent experiments) monocytes (**D**; 10 donors, 7 independent experiments) naïve PMNs (**G**; 7 donors, 3 independent experiments) and G-CSF/IFN-γ-stimulated PMNs (**J**; 7 donors, 3 independent experiments) towards opsonized target erythrocytes. **(B, E, H, K)** Titration curves of CRP dilutions ([100], 30, 10, 3, 1, 0 µg/ml) added to the cytotoxicity reaction with different anti-RhD IgG1 backgrounds ([1.0], 0.01, [0.003] and 0 µg/ml) using effector cells from the same donors as in **A, D, G, J**. **(C, F, I, L)** The impact of CRP addition ([100], 30, 10, 3, 1 µg/ml) on cytotoxicity triggered by (1.0), 0.01 and 0 µg/ml anti-RhD IgG1 using effector cells from different donors. All individual data points represent triplo data. Two-tailed paired t-test was used to determine significant differences. * $p \leq 0.05$; ** $p < 0.01$; N.S. not significant.

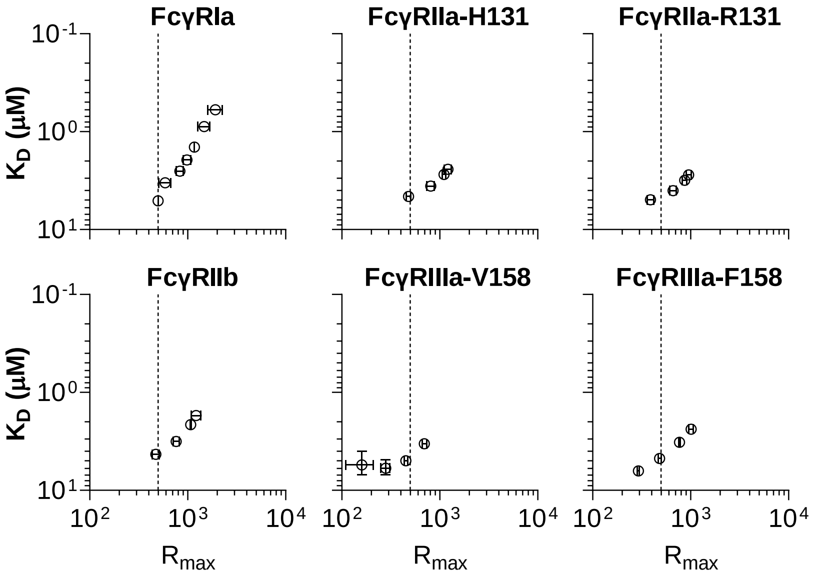

SUPPLEMENTAL FIGURE 13: FcR density effects on CRP affinity. Mean K_D values (μM ± S.E.M.; N.B. inversed log scale) of CRP-FcγR interactions as a function of the corresponding average R_{max} values (± S.E.M) which represent receptor spot density. Each data point represents averaged data of three independent experiments. Vertical dashed line indicate R_{max}= 500 where the exact affinity values reported in Figure 4 were calculated to.

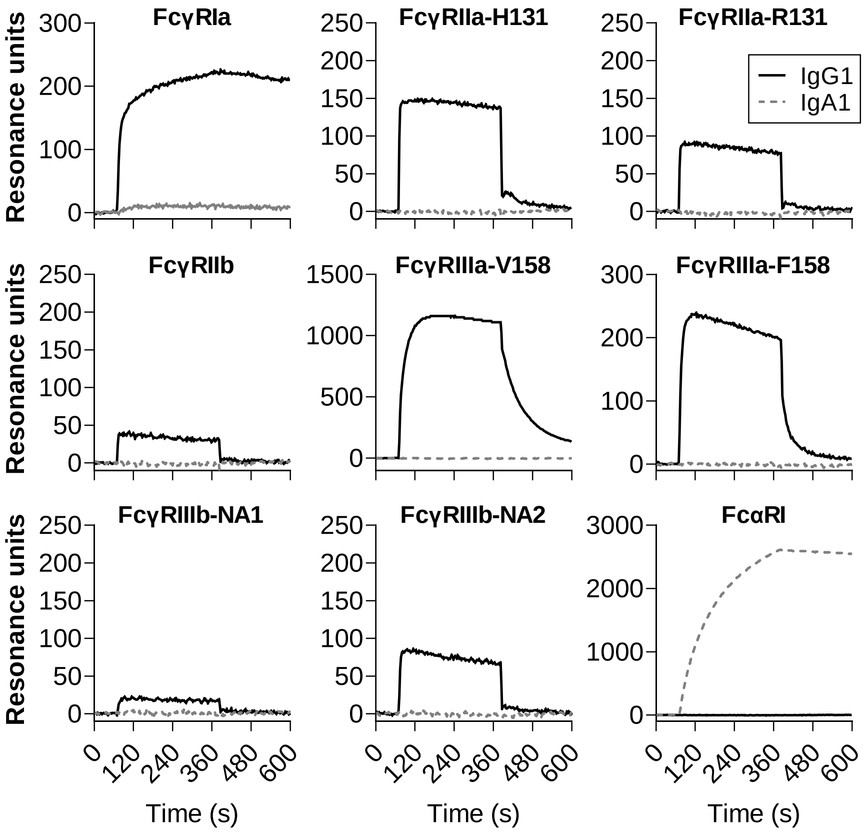

SUPPLEMENTAL FIGURE 14: IgG and IgA binding patterns towards immobilized FcRs. Representative sensorgrams showing binding kinetics of 1000 nM monoclonal human IgG1 (black lines) and IgA1 (grey lines) to C-terminally immobilized human FcγR classes and FcαRI. Binding was imaged in real-time and simultaneously.

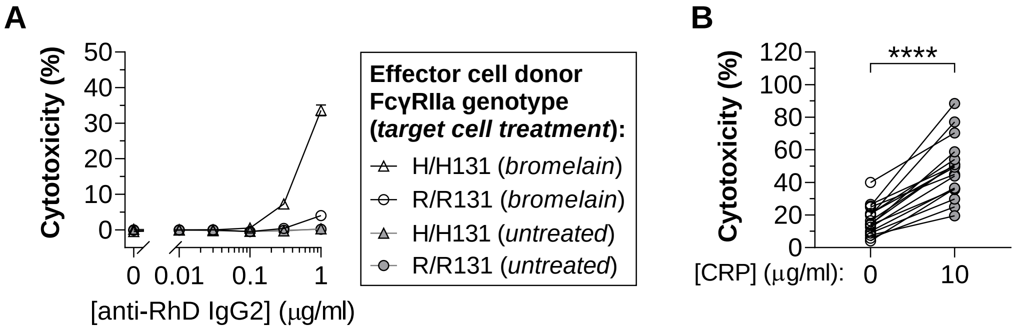

SUPPLEMENTAL FIGURE 15: CRP enhancement of IgG2-mediated cytotoxicity performed by activated PMNs. **(A)** Concentration curves of anti-RhD IgG2-mediated cytotoxicity (mean of triplo data ± S.E.M.) performed by G-CSF/IFN-γ-stimulated PMNs from FcγRIIa-genotyped donors (homozygous for H131, triangle, or R131, circles) and untreated (grey) or bromelain-treated (transparent) target erythrocytes. **(B)** IgG2 (0.5 μg/ml)-mediated ADCC responses in the presence or absence of 10 μg/ml CRP using G-CSF/IFN-γ-stimulated PMNs from 17 different FcγRIIa$^{H/H131}$ donors. Statistical differences have been determined by two-tailed paired t-test. **** $p < 0.0001$.

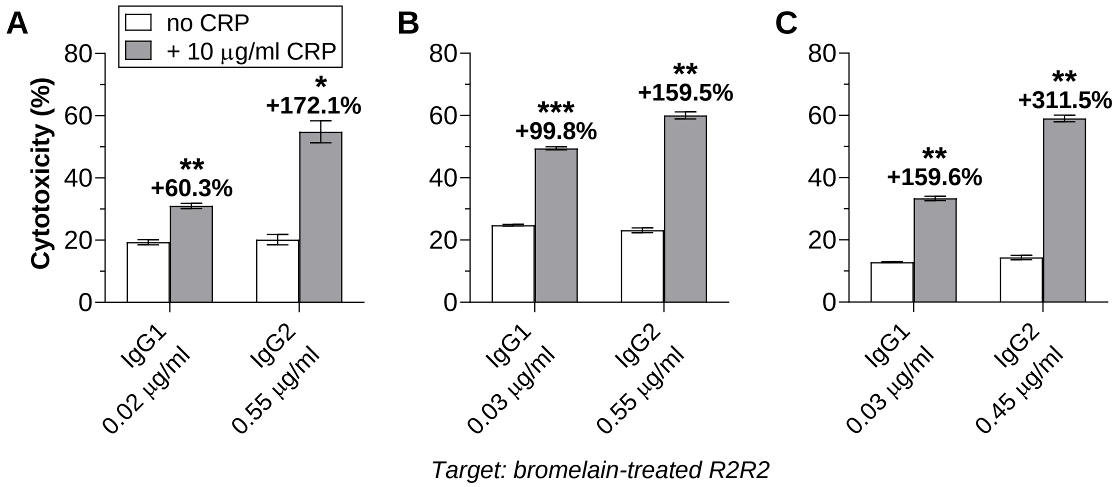

SUPPLEMENTAL FIGURE 16: CRP enhances IgG2-mediated baseline cytotoxicity consistently more efficiently than IgG1-mediated cytotoxicity performed by activated PMNs towards bromelain-treated erythrocytes. The effect of bromelain treatment on the magnitude of the CRP-enhancing effect on IgG-mediated cellular cytotoxicity (% lysed erythrocytes ± S.E.M., bars represent averages of triplo data) was assessed by using O/N G-CSF/IFN-γ-stimulated PMNs from three different FcγRIIa$^{H/H131}$ donors **(A-C)**. For each specific donor, target erythrocytes were treated with bromelain and incubated with effector cells in an E:T ratio of 2:1 in the presence of anti-RhD IgG1 or IgG2 with or without 10 µg/ml CRP. The CRP enhancement of IgG1- and IgG2-mediated cytotoxicity was only investigated for IgG1/2 concentrations (depicted under each panel) that elicited similar ADCC responses (~20% in **A**, ~25% in **B** and ~13% in **C**). Relative mean enhancement of baseline IgG1/2-mediated cytotoxicity by CRP is depicted in bold above each grey bar accompanied by significance compared to baseline signal. Each panel represents an independent experiment using G-CSF/IFN-γ-stimulated PMNs for one specific FcγRIIa$^{H/H131}$ donor. Significant differences were determined by two-tailed paired t-test. * $p \leq 0.05$; ** $p < 0.01$; *** $p < 0.001$.
